# Node-RADS: a systematic review and meta-analysis of diagnostic performance, category-wise malignancy rates, and inter-observer reliability

**DOI:** 10.1007/s00330-024-11160-1

**Published:** 2024-11-06

**Authors:** Jingyu Zhong, Shiqi Mao, Haoda Chen, Yibin Wang, Qian Yin, Qingqing Cen, Junjie Lu, Jiarui Yang, Yangfan Hu, Yue Xing, Xianwei Liu, Xiang Ge, Run Jiang, Yang Song, Minda Lu, Jingshen Chu, Huan Zhang, Guangcheng Zhang, Defang Ding, Weiwu Yao

**Affiliations:** 1 Department of Imaging, Tongren Hospital, Shanghai Jiao Tong University School of Medicine, Shanghai, 200336 China; 2 Department of Medical Oncology, Shanghai Pulmonary Hospital, Tongji University School of Medicine, Shanghai, 200433 China; 3 Department of General Surgery, Pancreatic Disease Center, Ruijin Hospital, Shanghai Jiao Tong University School of Medicine, Shanghai, 200025 China; 4 Department of Urology, Tongren Hospital, Shanghai Jiao Tong University School of Medicine, Shanghai, 200336 China; 5 Department of Pathology, Shanghai Sixth People’s Hospital, Shanghai Jiao Tong University School of Medicine, Shanghai, 200233 China; 6 Department of Dermatology, Shanghai Ninth People’s Hospital, Shanghai Jiao Tong University School of Medicine, Shanghai, 200011 China; 7 Department of Epidemiology and Population Health, Stanford University School of Medicine, Stanford, California 94305 USA; 8 Department of Biomedical Engineering, Boston University, Boston, Massachusetts 02215 USA; 9 Department of Pharmacovigilance, SciClone Pharmaceuticals (Holdings) Ltd., Shanghai, 200020 China; 10 MR Research Collaboration Team, Siemens Healthineers Ltd., Shanghai, 200126 China; 11 MR Application, Siemens Healthineers Ltd., Shanghai, 200126 China; 12 Department of Science and Technology Development, Ruijin Hospital, Shanghai Jiao Tong University School of Medicine, Shanghai, 200025 China; 13 Department of Radiology, Ruijin Hospital, Shanghai Jiao Tong University of Medicine, Shanghai, 200025 China; 14 Department of Orthopedics, Shanghai Sixth People’s Hospital, Shanghai Jiao Tong University School of Medicine, Shanghai, 200233 China

**Keywords:** Lymph nodes, Neoplasms, Magnetic resonance imaging, Tomography (X-ray computed), Systematic review

## Abstract

**Objective:**

To perform a systematic review and meta-analysis to estimate diagnostic performance, category-wise malignancy rates, and inter-observer reliability of Node Reporting and Data System 1.0 (Node-RADS).

**Methods:**

Five electronic databases were systematically searched for primary studies on the use of Node-RADS to report the possibility of cancer involvement of lymph nodes on CT and MRI from January 1, 2021, until April 15, 2024. The study quality was assessed by modified Quality Assessment of Diagnostic Accuracy Studies (QUADAS-2) and Quality Appraisal of Diagnostic Reliability (QAREL) tools. The diagnostic accuracy was estimated with bivariate random-effects model, while the pooled category-wise malignancy rates were obtained with random-effects model.

**Results:**

Six Node-RADS-CT studies and three Node-RADS-MRI studies covering nine types of cancer were included. The study quality was mainly damaged by inappropriate index test and unknown timing according to QUADAS-2, and unclear blindness during the rating process according to QAREL. The area under hierarchical summary receiver operating characteristic curve (95% conventional interval) was 0.92 (0.89–0.94) for Node-RADS ≥ 3 as positive and 0.91 (0.88–0.93) for Node-RADS ≥ 4 as positive, respectively. The pooled malignancy rates (95% CIs) of Node-RADS 1 to 5 were 4% (0–10%), 31% (9–58%), 55% (34–75%), 89% (73–99%), and 100% (97–100%), respectively. The inter-observer reliability of five studies was interpreted as fair to substantial.

**Conclusion:**

Node-RADS presented a promising diagnostic performance with an increasing probability of malignancy along higher category. However, the evidence for inter-observer reliability of Node-RADS is insufficient, and may hinder its implementation in clinical practice for lymph node assessment.

**Key Points:**

***Question*** *Node-RADS is designed for structured reporting of the possibility of cancer involvement of lymph nodes, but the evidence supporting its application has not been summarized.*

***Findings*** *Node-RADS presented diagnostic performance with AUC of 0.92, and malignancy rates for categories 1–5 ranged from 4% to 100%, while the inter-observer reliability was unclear.*

***Clinical relevance*** *Node-RADS is a useful tool for structured reporting of the possibility of cancer involvement of lymph nodes with high diagnostic performance and appropriate malignancy rate for each category, but unclear inter-observer reliability may hinder its implementation in clinical practice.*

## Introduction

The imaging evaluation of the likelihood of lymph node involvement is important in cancer staging as it is a substantial indicator of an adverse prognosis that usually has a great impact on clinical decision-making, especially in distinguishing surgically resectable cases from those that may benefit from non-surgical management [1]. The enlarged size of lymph nodes is a generally accepted criterion [2], while the heterogeneous measuring approach and various cutoff values for different anatomical sites can be confusing [3]. Another criterion is the configuration of lymph nodes with numerous descriptors, which can sometimes be helpful [4–7]. Further, there are attempts that combined size and configuration criteria to standardize the diagnostic workup for identifying positive lymph nodes in specific anatomic sites [8–11], but these approaches cannot be generalized to other disease entities and body sites.

**Fig. 1:**
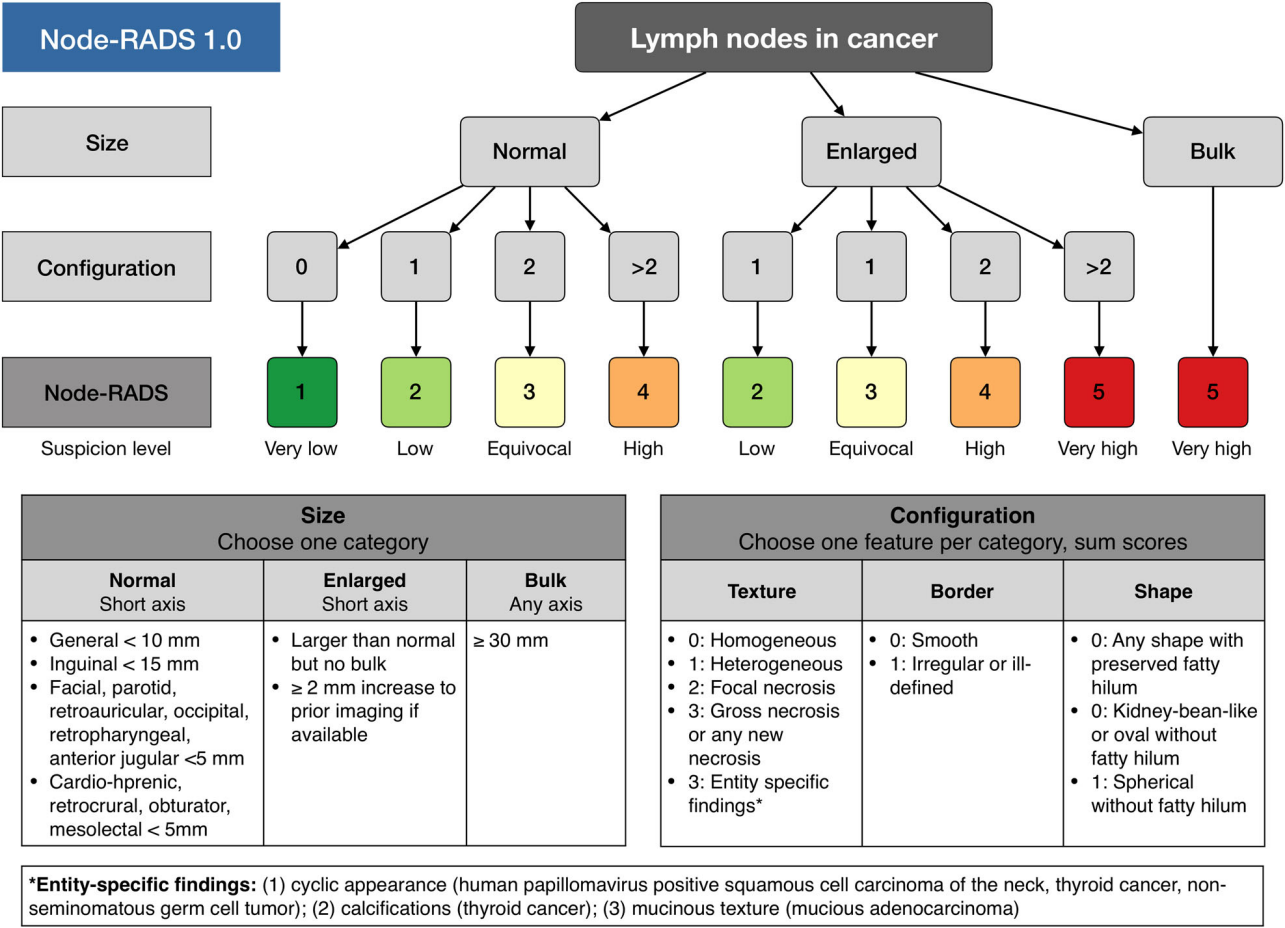
Node-RADS algorithm

Node Reporting and Data System 1.0 (Node-RADS) is therefore developed to standardize the reporting of radiologic assessment of lymph node involvement by cancer, irrespective of anatomical sites and primary tumors (Fig. 1) [12]. The Node-RADS evaluates the lymph nodes according to size (normal, enlarged, or bulk) and configurations (texture, border, and shape). Then, the lymph nodes are scored from 1 to 5 to reflect the possibility of malignancy: 1 (very low), 2 (low), 3 (equivocal), 4 (high), and 5 (very high). This scoring system aims to allow a standardized reporting of lymph node involvement on oncological CT and MRI scans with a RADS-style approach. It is expected to help clinical referrers to more easily make treatment decisions by describing the likelihood of cancer involvement with straightforward definitions.

Although the Node-RADS has the potential to facilitate uniformity in reporting possible cancer involvement of lymph nodes on CT and MR scans, there is currently no evidence supporting its accuracy and reliability in clinical practice. It is necessary to evaluate the diagnostic performance of Node-RADS and ensure that category-wise malignancy rates are appropriate, to avoid misclassifications [13–22]. Further, it is also important to investigate whether the Node-RADS has a high inter-observer reliability to decrease the variability gap in lymph node involvement reporting among radiologists [23–25].

Therefore, this study aimed to perform a systematic review and meta-analysis to estimate diagnostic performance, category-wise malignancy rates, and inter-observer reliability of Node-RADS.

## Methods

### Study protocol and workflow

We have drafted a study protocol before conduction of our study (Supplementary Note S1), and registered it via PROSPERO, the International Prospective Register of Systematic Reviews (https://www.crd.york.ac.uk) [26], with an identifier of CRD 42024534540. We reported the study according to Preferred Reporting Items for Systematic reviews and Meta-Analyses (PRISMA) statements [27–30]. We conducted the literature search, study selection, data extraction, and quality assessment by two of three independent reviewers with 6 years of experience in radiology, medical oncology, and general surgery, respectively. We performed the data analysis by one reviewer supervised by a biostatistical expert. The disagreements were solved with discussions or consultation with the review group consisting of health professionals with diverse background and experience.

### Literature search and study selection

We systematically searched five electronic databases, namely PubMed, Embase, Web of Science, China National Knowledge Infrastructure, and Wanfang Data, for primary studies on the use of Node-RADS to report the possibility of cancer involvement of lymph nodes on CT and MRI. We included all the available databases as possible to reduce the selection bias of our systematic review and to maximize the likelihood of finding relevant studies [29]. The time of publication was restricted between January 1, 2021, and April 15, 2024, as the Node-RADS was proposed in early 2021 [12]. The language of publication was restricted to English, Chinese, Japanese, German, and French. The titles and abstracts of records were screened after the removal of duplications. The full texts and supplementary materials of the potential records were then retrieved for eligibility evaluation. The reference lists of included studies and relevant reviews were browsed to identify additional eligible studies. The details of the search strategy and study selection are provided (Supplementary Note S2).

### Data extraction and quality assessment

We have developed a tool to standardize the data extraction process of our review (Supplementary Table S1). The data extraction sheet included bibliographic information, methodological details, patient characteristics, imaging protocol, rating process, and diagnostic performance metrics. For diagnostic performance and category-wise malignancy rates studies, we assessed their quality per modified Quality Assessment of Diagnostic Accuracy Studies (QUADAS-2) [31] with specified signal questions for this review (Supplementary Table S2). This tool assessed the risk of bias and applicability concerns in terms of patient selection, index test, reference standard, flow and timing. For inter-observer reliability studies, we further evaluated their quality per Quality Appraisal of Diagnostic Reliability (QAREL) [32] according to the guidance for answering tool items (Supplementary Table S3). This tool evaluated the eleven items that explore seven principles covering the spectrum of subjects, spectrum of examiners, examiner blinding, order effects of examination, suitability of the time interval among repeated measurements, appropriate test application and interpretation, and appropriate statistical analysis. These tools have been discussed before the formal data extraction and quality assessment. The items that have been discussed are recorded (Supplementary Note S3).

### Data synthesis and analysis

We conducted data analysis using Stata version 15.1 (https://www.stata.com) and R language version 4.2.1 (https://www.r-project.org/) within RStudio version 1.3.1093 (https://posit.co/) with relevant packages, and a website Application (https://www.metaumbrella.org) [33–36]. Continuous variables were described as mean ± standard deviation, median (range), while categorical variables were described as proportion (percentage). The data for meta-analysis were extracted directly from the articles or reconstructed using available data. The diagnostic accuracy was estimated with the bivariate random-effects model, while the category-wise malignancy rates were obtained with the random-effects model. The pooled inter-observer reliability was not available due to insufficient data. The Node-RADS document suggested to report Node-RADS 1 and 2 as negative nodes and Node-RADS 4 and 5 as positive nodes. However, the Node-RADS 3 should be reported depending on the stage and histologic grade of the primary tumor [12]. Therefore, we applied two cutoffs for positive events: Node-RADS ≥ 3 and Node-RADS ≥ 4 for the diagnostic performance analysis. The two-by-two tables for each cutoff were extracted or reconstructed, respectively. The data of overall diagnostic performance were chosen if reported. Otherwise, the data of the observer with worst diagnostic performance were chosen, to provide a conservative estimate for Node-RADS. The hierarchical summary receiver operating characteristic (HSROC) curve was plotted. The sensitivity, specificity, positive predictive value, negative predictive value, and diagnostic odds ratio were calculated with and their 95% confidence interval (CI) and corresponding *p-*value. The heterogeneity was estimated by the Cochran’s *Q* and the Higgins *I*^*2*^ statistic. We did not conduct *post hoc* subgroup analysis or meta-regression to investigate the potential source of heterogeneity due to the limited number of included studies. The small study effects were assessed by Egger’s test, and Begg’s test. The publication bias was assessed using Deeks’ funnel test and trim and fill method analysis. The 95% prediction interval and the excess significance bias were conducted. A two-tailed *p*-value < 0.05 was considered as statistically significant, while a two-tailed *p*-value < 0.10 indicated a high risk of publication bias. The evidence level for supporting Node-RADS in clinical practice is rated according to the results of meta-analyses (Supplementary Table S4) [35]. The details of data synthesis and analysis are provided (Supplementary Note S4).

## Results

### Study inclusion

**Fig. 2:**
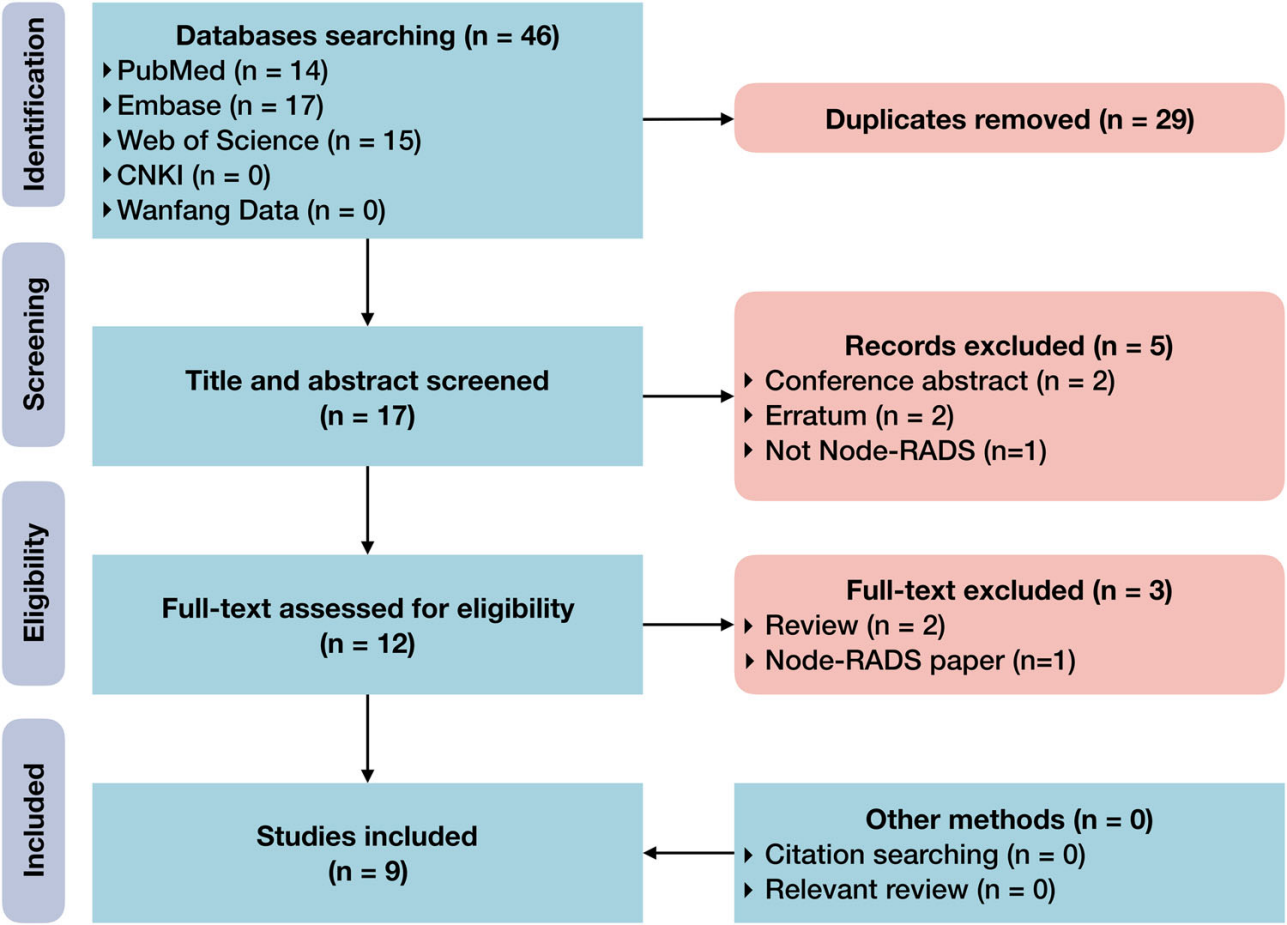
Diagram of study inclusion flow

The process of study inclusion is presented (Fig. 2). The initial literature search resulted in 46 records, and 29 of them were duplications. We screened titles and abstracts of 17 records. The retrieved full-texts and supplementary materials of twelve studies were evaluated for eligibility. Finally, nine studies were included for analysis [37–45]. We did not identify additional eligible studies by browsing the reference lists of included studies or relevant reviews. The included studies and excluded full texts with justifications are recorded (Supplementary Note S2).

### Study characteristics

The characteristics of the nine included studies are presented (Table 1). The studies were performed in Germany [39, 40, 43], Italy [38, 41, 42], China [44, 45], and Switzerland [37], respectively. The studies covered nine different cancer types, including melanoma [37], bladder cancer [38], cholangiocarcinoma [39], gastric cancer [40], prostate cancer [41], colon cancer [42], lung cancer [43], cervical cancer [44], and nasopharyngeal carcinoma [45], respectively. The studies were more frequently published in clinical journals (5/9, 56%) [37–39, 41, 45]. All the studies were performed with a retrospective design (9/9, 100%) [37–45] within a single center (9/9, 100%) [37–45]. The mean ± standard deviation, median (range) of number of patients and percentage of positive events were 86.8 ± 37.4, 91.0 (25.0–150.0), and 37.8 ± 17.3%, 40.7% (6.5–69.0%), respectively. More studies were conducted with CT (6/9, 67%) [37–40, 42, 43] than with MRI (3/9, 33%) [41, 44, 45]. Node-RADS were more frequently rated by two observers (6/9, 67%) [39, 40, 42–45] according to the original Node-RADS algorithm (8/9, 89%) [37–45]. The mean ± standard deviation, median (range) year of observer experience was 9.3 ± 6.8, 8.0 (2.0–25.0). The study characteristics for each study are summarized (Supplementary Tables S5–S8).

**Table 1:** Characteristics of included studies

Study characteristics	Data
Journal type (*N* = 9), *n* (%)	
Radiological journal	4 (44)
Clinical journal	5 (56)
Country (*N* = 9), *n* (%)	
Germany	3 (33)
Italy	3 (33)
China	2 (22)
Switzerland	1 (11)
Study design (*N* = 9), *n* (%)	
Retrospective	9 (100)
Prospective	0 (0)
Study center (*N* = 9), *n* (%)	
Single center	9 (100)
Multiple centers	0 (0)
Cancer type (*N* = 9), *n* (%)	
Bladder cancer	1 (11)
Cervical cancer	1 (11)
Cholangiocarcinoma	1 (11)
Colon cancer	1 (11)
Gastric cancer	1 (11)
Lung cancer	1 (11)
Melanoma	1 (11)
Nasopharyngeal carcinoma	1 (11)
Prostate cancer	1 (11)
Imaging modality (*N* = 9), *n* (%)	
MRI	3 (33)
CT	6 (67)
Number of patients, mean ± standard deviation, median (range)	86.8 ± 37.4, 91.0 (25.0–150.0)
Number of total events, mean ± standard deviation, median (range)	110.9 ± 63.5, 91.0 (49.0–216.0)
Number of positive events, mean ± standard deviation, median (range)	40.6 ± 38.6, 35.0 (14.0–140.0)
Percentage of positive events, mean ± standard deviation, median (range)	37.8 ± 17.3%, 40.7% (6.5–69.0%)
Number of observers (*N* = 9), *n* (%)	
One observer	3 (33)
Two observers	6 (67)
Year of experience of observers, mean ± standard deviation, median (range)	9.3 ± 6.8, 8.0 (2.0–25.0)
Node-RADS algorithm (*N* = 9), *n* (%)	
Original Node-RADS	8 (89)
Modified Node-RADS	1 (11)

*Node-RADS* Node Reporting and Data System 1.0

### Study quality

**Fig. 3:**
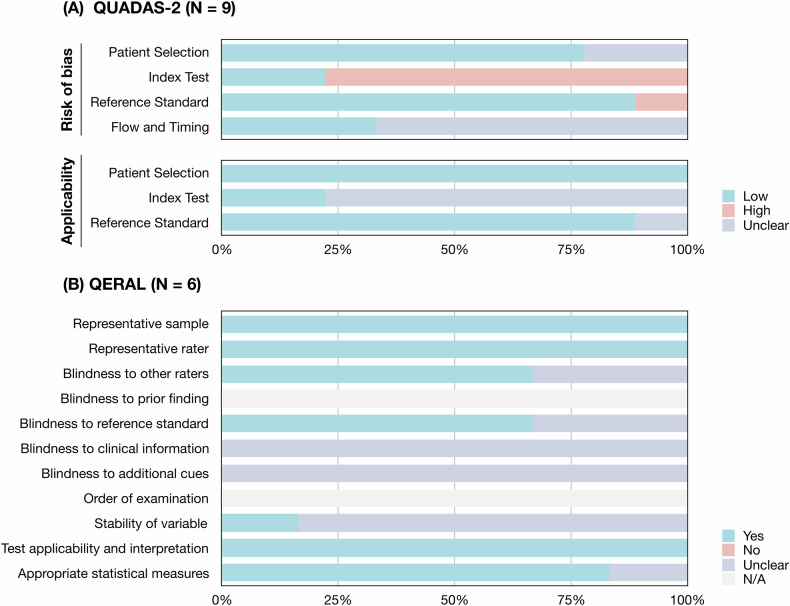
QUADAS-2 and QAREL assessment of included studies. **A** The QUADS-2 assessment of 9 included studies that reported the diagnostic performance and category-wise malignancy rates. **B** The QAREL assessment of 6 included studies that reported inter-observer reliability

The results of study quality assessments are presented (Fig. 3). Nine studies of diagnostic performance and category-wise malignancy rates were assessed using the QUADAS-2 tool [37–45]. The main risk of bias and application concerns were mainly related to index test and flow and timing due to incomplete imaging protocol [37, 39–41, 43–45], inappropriate rating process [37, 38, 41], and the unknown interval between index test and reference standard [37, 38, 40, 41, 44, 45]. Six inter-observer reliability studies were evaluated using the QAREL tool [39, 40, 42–45]. The disadvantages of these studies were unclear blindness during the rating process [39, 40, 42–45]. The QUADAS-2 and QAREL assessment of each study by two reviewers is recorded (Supplementary Tables S9, S10).

### Meta-analysis

**Fig. 4:**
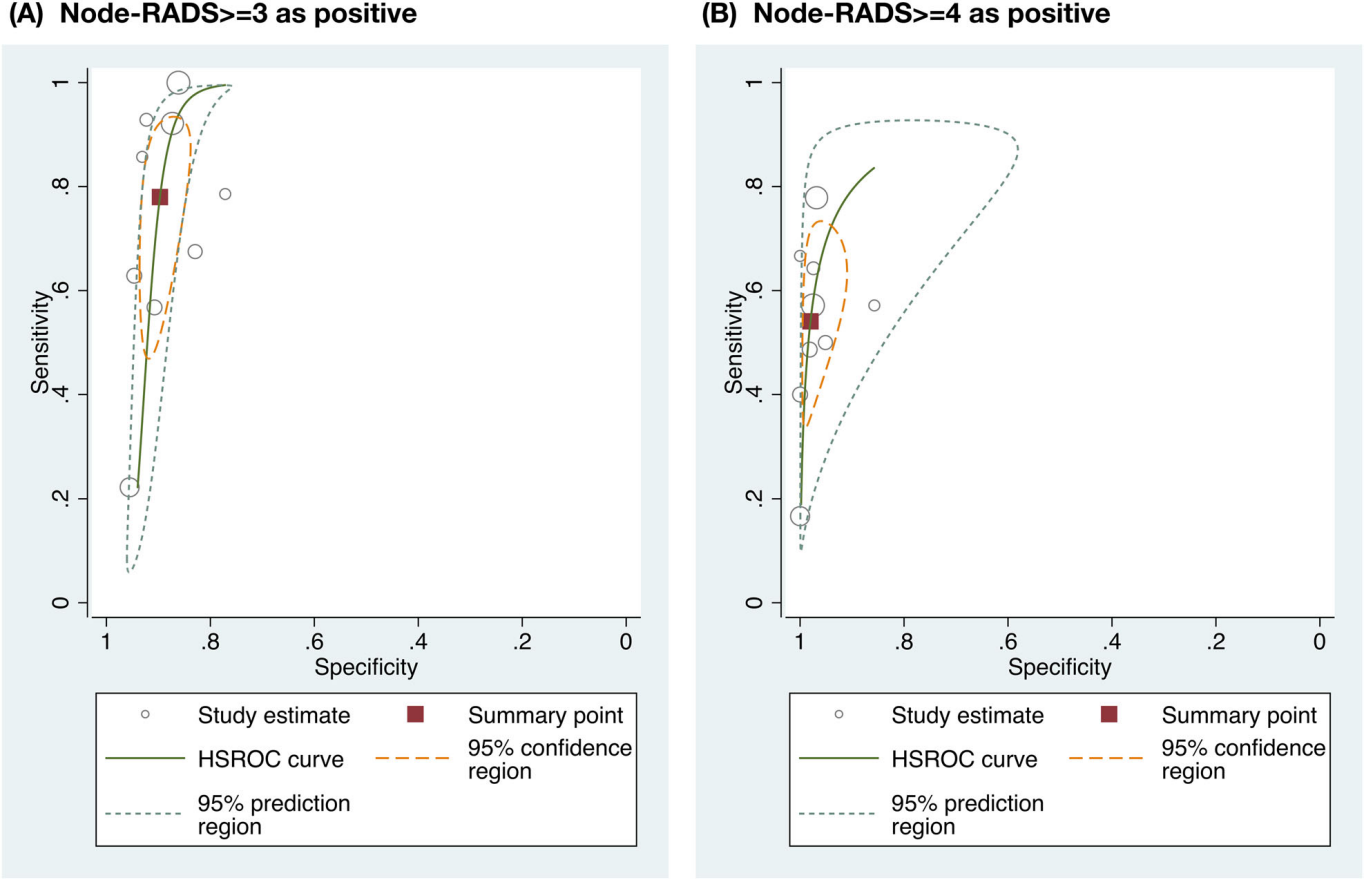
Hierarchical summary receiver operating characteristic curve. **A** The area under curve for diagnostic performance of positive lymph nodes when Node-RADS ≥ 3 was considered as positive. **B** The area under curve for diagnostic performance of positive lymph nodes when Node-RADS ≥ 4 was considered as positive

**Fig. 5:**
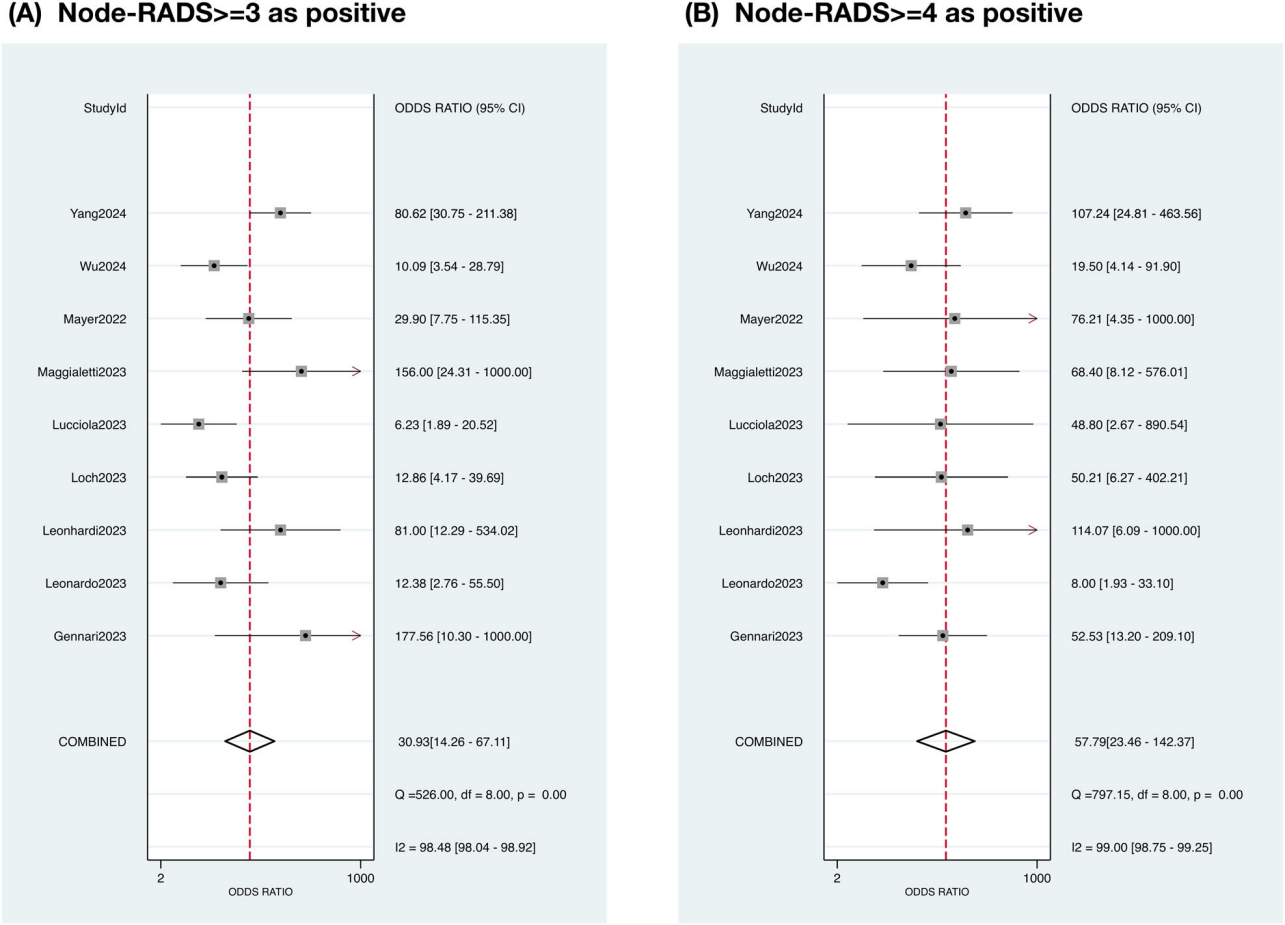
Forest plot of pooled diagnostic odds ratio. **A** The diagnostic performance of positive lymph nodes when Node-RADS ≥ 3 was considered as positive. **B** The diagnostic performance of positive lymph nodes when Node-RADS ≥ 4 was considered as positive

All the data for meta-analysis are directly extracted or reconstructed using available data (Supplementary Tables S11, S12). The diagnostic performance of Node-RADS was pooled using data from nine included studies (Supplementary Figs. S1, S2) [37–45]. The areas under the HSROC curve (95% CI) were 0.92 (0.89–0.94) for Node-RADS ≥ 3 as positive, and 0.91 (0.88–0.93) for Node-RADS ≥ 4 as positive, respectively (Fig. 4). The diagnostic odds ratios (95% CI) were 30.93 (14.26–67.11) for Node-RADS ≥ 3 as positive, and 56.79 (23.46–142.37) for Node-RADS ≥ 4 as positive, respectively (Fig. 5). The heterogeneity for the meta-analyses was high. The significant difference between the 95% confidence region and the 95% prediction region was large, which also indicated a high possibility of heterogeneity across the studies. No small study effects were identified by Egger’s test or Begg’s test. No publication bias was found using Deeks’ funnel test or trim and fill analysis. The 95% prediction interval excluded the null value. No excess significance bias was identified. The evidence level for supporting Node-RADS in clinical practice is rated as weak, mainly due to insufficient positive events (Table 2). The category-wise malignancy rates were pooled using data from eight included studies (Table 3) [37–39, 41–45]. The pooled malignancy rates (95% CIs) of Node-RADS 1, 2, 3, 4, and 5 were 4% (0–10%), 31% (9–58%), 55% (34–75%), 89% (73–99%), and 100% (97–100%), respectively (Supplementary Fig. S3). The heterogeneity in pooled analysis of malignancy rates of Node-RADS 1 to 4 was considered to be high, but not in that of Node-RADS 5. The meta-analysis for inter-observer reliability for meta-analysis was not available due to insufficient data (Supplementary Table S13). The reported inter-observer reliability using kappa statistics ranged from 0.35 to 0.86 which were interpreted as fair to substantial.

**Table 2:** Evidence level for clinical application of Node-RADS

Diagnostic performance	Node-RADS ≥ 3 as positive	Node-RADS ≥ 4 as positive
Sample size		
Number of studies	9	9
Number of total events	998	998
Number of positive events	365	365
Meta-analysis results		
DOR (95% CI)	30.93 (14.26–67.11)	56.79 (23.46–142.37)
*p*-value for DOR	9.15 × 10^−^^17^	1.25 × 10^−^^23^
Sensitivity (95% CI)	0.78 (0.59–0.90)	0.54 (0.41–0.67)
Specificity (95% CI)	0.90 (0.86–0.92)	0.98 (0.95–0.99)
Positive likelihood ratio (95% CI)	7.59 (5.83–9.88)	27.08 (10.97–66.84)
Negative likelihood ratio (95% CI)	0.25 (0.13–0.48)	0.47 (0.35–0.62)
Aera under curve (95% CI)	0.92 (0.89–0.94)	0.91 (0.88–0.93)
Cochran’s *Q* test, Q (*p*-value)	53.30 (*p* < 0.001)	16.44 (*p* < 0.001)
Higgins *I*^*2*^ test, estimate (95% CI)	96% (93– 99%)	88% (75–100%)
Egger’ s test, *p*-value	0.314	0.335
Begg’ s test, *p*-value	0.348	0.602
Deeks’ test, *p*-value	0.933	0.150
DOR (95% CI) after trim and fill method	20.46 (9.34–44.83)	28.10 (14.72–53.63)
95% PI, excluding the null	2.50–301.46, Yes	8.57–188.52, Yes
DOR of largest study, excluding the null	10.30–3060.04, Yes	13.20–209.10, Yes
Excess significance	No	No
Level of evidence according to Ioannidis criteria		
Evidence level	Weak	Weak
Main weakness	Insufficient positive events	Insufficient positive events

*Node-RADS* Node Reporting and Data System 1.0, *CI* confidence interval, *DOR* diagnostic odds ratio

**Table 3:** The pooled category-wise malignancy rates

Node-RADS cetology	Positive events	Total events	Estimate (95% CI)	Cochran’s *Q* test, Q (*p*-value)	Higgins *I*^*2*^ test, estimate
Node-RADS-1	20	422	4% (0–10%)	34.29 (*p* < 0.001)	79.58%
Node-RADS-2	53	144	31% (9–58%)	71.37 (*p* < 0.001)	90.19%
Node-RADS-3	54	102	55% (34–75%)	25.80 (*p* < 0.001)	72.86%
Node-RADS-4	91	104	89% (73–99%)	20.14 (*p* = 0.01)	65.25%
Node-RADS-5	110	113	100% (97–100%)	8.21 (*p* = 0.22)	26.96%

*Node-RADS* Node Reporting and Data System 1.0, *CI* confidence interval

## Discussion

Our study found that Node-RADS presented a promising diagnostic performance for both cutoff values of Node-RADS ≥ 3 as positive and Node-RADS ≥ 4 as positive. However, it is still unclear which one is more appropriate to be used as the cutoff to indicate the cancer involvement of lymph nodes. The probability of malignancy increased with the Node-RADS category, as it is designed to be an imaging system that incorporates a Likert-type scale for the likelihood of malignant nodal disease. Nevertheless, the evidence for inter-observer reliability of Node-RADS is insufficient. The current heterogeneous data cannot support its implementation as a routine clinically practicable tool for lymph node assessment.

Our review showed room for improvement in the methodological aspect of Node-RADS studies. First, the Node-RADS study should be designed with caution. Our review included both CT and MRI studies on nine cancer types. Unfortunately, all of them were retrospective single-center studies with a largest sample size of 150 patients. It is unclear whether the cancer type influences the diagnostic performance, category-wise malignancy rates, and inter-observer reliability. It would be necessary to summarize the results according to specific cancer type when prospective, multicenter, large sample studies are available. It allows the Node-RADS to be better interpreted with TNM (Tumor, nodes, metastasis) staging system [1] and RECIST (Response Evaluation Criteria in Solid Tumors) criteria [46–48]. Second, the details of the rating process should be better reported. The assessment of diagnostic performance studies by QUADAS-2 indicated risk of bias and application concerns in terms of index test and flow and timing. Our review found that the acquisition and reconstruction details were underreported. However, they are not necessary to be consistent to facilitate the broad applicability of Node-RADS. The Node-RADS does not rely on specific technical requirements. Only contrast enhancement is mandatory for CT scans to assess the configuration categories [12]. The variations in imaging modality may not have a large impact on the size measurement and configuration characterization. Nevertheless, the settings during the rating process may have a potential influence on the results and may harm the generalizability of Node-RADS. It is also unclear how short the interval between imaging and surgery is appropriate in Node-RADS investigations. Third, inter-observer reliability should be emphasized. Although Node-RADS in six studies were rated by two observers, the inter-observer reliability was underreported. The assessment of inter-observer reliability studies by QAREL suggested obvious disadvantages in blindness. Many studies reported the blindness to other observers and reference standards, but the blindness to clinical information and additional cues was frequently neglected. A complete reporting of inter-observer reliability is encouraged to allow future quantitative estimation of this topic and identification of room for improvement [49–51]. We believe these methodological disadvantages should be avoided in future studies to allow more generalizable and high-quality evidence to support the application of Node-RADS.

There are shortcomings of the Node-RADS system identified during meta-analysis. First, although the meta-analysis on the Node-RADS studies showed promising performance of Node-RADS, the cutoff for the Node-RADS has not been determined. It is suggested that the Node-RADS-3 should be interpreted according to the histological type of the primary cancer [12]. Our study found that both cutoffs of Node-RADS ≥ 3 and Node-RADS ≥ 4 exhibit high diagnostic performance, but the specificity of Node-RADS ≥ 3 is much higher than Node-RADS ≥ 4 at the expense of a little decrease in sensitivity. Therefore, we considered that Node-RADS ≥ 3 may be an optimal cutoff for Node-RADS if there is no specific indication. Second, there is no pre-defined malignancy rate for each Node-RADS category [12]. Our review showed a malignancy rate that escalates from 4% to 100% with increasing Node-RADS category. It is necessary to provide predicted category-wise malignancy rates to allow the radiologists to appropriately assign the Node-RADS category. Thereby, the clinicians can later correctly interpret the Node-RADS category and make the optimum treatment decision. Moreover, it is a crucial topic to evaluate the misclassification rate in each category compared to the predicted in the validation study [52, 53]. Different causes behind the misinterpretation errors can be identified through reviews to allow improvement of this tool [54, 55]. Third, the level of reporting has not been clearly stated in the study. It is suggested to report the node with the highest category if there are multiple abnormal nodes in a specific nodal group, unless the number of lymph node metastases influences staging or treatment decision [12]. As a result, the included studies reported the Node-RADS sometimes per patient [38, 40–44] and sometimes per lymph node [37, 39, 45]. It seemed that the selection of the reporting level is relatively casual as there is no illustration of the selection of the reporting level. This should be stated more clearly when revising the Node-RADS system. Forth, one of the main aims of Node-RADS is to provide the best possible assessment of the nodal status of patients with consistency. However, the data for inter-observer reliability of Node-RADS was currently not sufficient. The previous studies have reported fair to substantial agreement of Node-RADS between observers who have 2 to 25 years of experience. Achieving a high level of agreement between observers is the prerequisite for a tool to increase the diagnostic performance of observers with less experience. We believe it is necessary to report and find whether there are factors that damage the inter-observer reliability, and provide corresponding training for future generalization [23–25].

The Node-RADS currently has not been widely accepted by the radiological community. According to the step‐by‐step structured and systematic approach methodology, the clinical application of Node-RADS should pass six steps, including validation of technical performance, validation of diagnostic performance, validation of diagnostic impact, validation of therapeutic impact, patient outcomes, and finally, societal impact [56]. The Node-RADS seems to be still in the second step. Currently, the evidence for using the Node-RADS per cancer type is limited. Neither the diagnostic performance nor the inter-observer reliability is clear. There is no well-organized large-scale multicenter study on Node-RADS, while this knowledge should be provided to allow radiologists and clinicians to use this tool with confidence. Further, the relation between the Node-RADS and other RADS systems should be declared, especially those that have special considerations on the lymph nodes [9–11, 57]. We consider that the Node-RADS can mainly serve as an optimal tool for assessing the likelihood of malignancy of the lymph nodes, when there is no specific tool available. In contrast, if there is a RADS system that evaluates the original disease and lymph nodes as a whole, the specific tool may provide a more accurate diagnosis for the situation. Moreover, the limited application of Node-RADS can also be attributed to insufficient efforts in the promotion. Unlike other RADS systems that were developed and promoted by the American College of Radiology [58], only a single article is published to introduce the Node-RADS [12] along with several validation studies [37–45]. More efforts on the promotion may be necessary for the wide acceptance of this system. Nevertheless, we believe that the Node-RADS will gain popularity in the future. The example of Ovarian-adnexal Reporting and Data System (O-RADS) MRI may serve as a reference for Node-RADS to translate the current radiological research into a clinical application tool [59].

Our study has several limitations. First, our review included a limited number of studies. It can be attributed to the fact that the Node-RADS has not been widely accepted in clinical routine as other RADS. However, this review provides a timely summary of the current evidence to support the clinical application of this tool. We believe this review allows more radiologists to know and apply this tool. Second, the included studies were performed in nine different cancer types. Therefore, the results of our review should be interpreted with caution regarding the extrapolation of these data to other cancer types. Nevertheless, we consider that the Node-RADS needs to be validated in multiple cancer types, as this scoring system is designed to have broad applicability to cancer types across multiple anatomic sites [12]. Third, we were unable to quantitively estimate the inter-observer reliability of Node-RADS due to insufficient data. A high inter-observer reliability is necessary for this scoring system to serve as a tool to improve the consistency of reporting of lymph nodes and promote communications with referring clinicians. We will perform a meta-analysis if there are more studies on the inter-observer reliability of Node-RADS with complete reporting. Fourth, we did not conduct subgroup analysis or meta-regression to investigate the potential source of heterogeneity in pooled diagnostic performance and category-wise malignancy rates. We considered that the current number of included studies did not allow us to reach robust results by *post hoc* analysis. We will explore whether the cancer type, imaging modality, observer experience, and other factors contribute to heterogeneity when there are more studies available. Finally, the quality assessment of included studies indicated a high risk of bias. The conclusion of our review should therefore be interpreted with caution. Further validation study is needed to confirm whether the Node-RADS is with appropriate accuracy and reliability across different cancer types.

In conclusion, Node-RADS is a useful tool for structured reporting for the possibility of cancer involvement of lymph nodes, demonstrating high diagnostic performance with appropriate malignancy rate for each category, while unclear inter-observer reliability may hinder its implementation in clinical practice. Future studies are encouraged to further validate and completely report the diagnostic performance, category-wise malignancy rates, and inter-observer reliability of Node-RADS to allow wider application of this tool in clinical routine.

## Supplementary information

ELECTRONIC SUPPLEMENTARY MATERIAL

## Abbreviations

CI: Confidence interval
HSROC: Hierarchical summary receiver operating characteristic
Node-RADS: Node Reporting and Data System 1.0
QAREL: Quality appraisal of diagnostic reliability
QUADAS-2: Modified quality assessment of diagnostic accuracy studies
