# Supplementary material for: Node-RADS: a systematic review and meta-analysis of diagnostic performance, category-wise malignancy rates, and inter-observer reliability
Source: Eur Radiol. 2024 Nov 6;35(5):2723–35. doi: 10.1007/s00330-024-11160-1 (PMC12021726; doi:10.1007/s00330-024-11160-1)

Node-RADS: a systematic review and meta-analysis of diagnostic performance, category-wise malignancy rates, and inter-observer reliability
ELECTRONIC SUPPLEMENTARY MATERIAL

List of Supplementary Materials

Supplementary Note S1 Study protocol
Supplementary Note S2 Search strategy and study selection
Supplementary Note S3 Data extraction and quality assessment
Supplementary Note S4 Data synthesis and analysis

Supplementary Table S1 Data extraction tool
Supplementary Table S2 QUADAS-2 tool for diagnostic accuracy study with modified questions
Supplementary Table S3 QAREL tool for diagnostic reliability study with consensus on items
Supplementary Table S4 Category of five levels of evidence based on meta-analyzes

Supplementary Table S5 Methodological aspect of included studies
Supplementary Table S6 Patient characteristics of included studies
Supplementary Table S7 Imaging protocol of included studies
Supplementary Table S8 Rating process of included studies

Supplementary Table S9 QUADAS-2 assessment by two reviewers and consensus results
Supplementary Table S10 QAREL assessment by two reviewers and consensus results

Supplementary Table S11 Two-by-two data for meta-analysis
Supplementary Table S12 Category-wise malignancy rate for meta-analysis
Supplementary Table S13 Inter-observer reliability for meta-analysis

Supplementary Figure S1 Meta-analysis of diagnostic performance for Node-RADS ≥ 3 as positive
Supplementary Figure S2 Meta-analysis of diagnostic performance for Node-RADS ≥ 4 as positive
Supplementary Figure S3 Forest plots of pooled category-wise malignancy rates

Eur Radiol (2024) Zhong JY, Mao SQ, Chen HD, et al.

Supplementary Note S1 Study protocol

This systematic review has been registered via the International prospective register of systematic reviews (PROSPERO; https://www.crd.york.ac.uk/prospero/). The following is the details of the registration.

First draft date: 09 Apr 2024
Last edit date: 15 Apr 2024
PROSPERO ID: 534540/ CRD 42024534540

Review title
Node-RADS: a systematic review and meta-analysis of diagnostic performance, category-wise malignancy rates, and inter-observer reliability

Review question
Is Node Reporting and Data System 1.0 (Node-RADS) clinically practicable with diagnostic accuracy and reliability?

Searches
Our search will include the following electronic databases: PubMed, Embase, Web of Science, China National Knowledge Infrastructure, and Wanfang Data. The study should be published after 2021, since the Node-RADS was proposed on 2021. The study should be published in English, Chinese, Japanese, German, or French, to allow detailed assessment by our group. The formal search string will be developed by a radiologist with experience in systematic review.

Condition or domain being studied
Node-RADS systematically classifies the degree of suspicion of lymph node involvement based on the synthesis of established imaging findings. Straightforward definitions of imaging findings for two proposed scoring categories "size" and "configuration" are combined into assessment categories between 1 ("very low likelihood") and 5 ("very high likelihood"). It is expected to improve communication with referring physicians and promote the consistency of reporting for primary staging and in response assessment settings. However, it is still unclear whether Node-RADS is clinically practicable with diagnostic accuracy and reliability.

Participants/population
Participants' inclusion criteria:
1) patients with histologically confirmed cancer at any anatomical site;
2) patients had undergone CT or MRI scan for assessment;
3) patients with Node-RADS ratings for lymph nodes;
4) patients with histological results for malignancy lymph nodes, and histological results or composite clinical reference standard for benign lymph nodes.

Participants' exclusion criteria:
1) not human patients, e.g., cell line, xenotransplant;
2) not histologically confirmed cancer, e.g., benign diseases, suspected cancer without histological evidence;
3) with imaging modalities other than CT or MRI scan, e.g., ultrasound, radiography;
4) Node-RADS rating not given;
5) without histological results or composite clinical reference standard of the assessed lymph nodes.

Intervention(s), exposure(s)
Node-RADS rating on CT or MRI scans.

Comparator(s)/control
Standard-of-care imaging.

Types of study to be included
Studies describing the diagnostic accuracy and/or reliability of Node-RADS based on contrast-enhanced CT or MRI will be included in this review. The studies will be included in systematic review if they are with full-text available and sufficient information for assessing the methodological quality. The studies will be included in meta-analysis if they are sufficient data of diagnostic accuracy and/or reliability.

Study inclusion criteria:
1) studies are reported in English, Japanese, Chinese, German or French with institutional full-text availability;
2) the cohort consists of patients with confirmed cancer at any anatomical site;
3) patients had undergone CT or MRI for Node-RADS assessment;
4) with histological results for malignancy lymph nodes, and histological results or composite clinical reference standard

for benign lymph nodes.

Study exclusion criteria:
1) duplicate studies;
2) studies published before 2021;
3) reviews, technical reports, letters to editors, comments to published studies, conference proceedings, case reports, brief communications and articles with insufficient information for assessing the methodological quality.

Main outcome(s)
The characteristics of included study will be summarized. The diagnostic accuracy and reliability of Node-RADS will be summarized. The methodological quality will be assessed.

Measures of effect
The diagnostic accuracy and reliability of Node-RADS will be firstly descriptively summarized. The studies with diagnostic accuracy results will be assessed using modified Quality Assessment of Diagnostic Accuracy Studies (QUADAS-2) tool for risk of bias and concern on application. The studies with diagnostic reliability results will be assessed using Quality Appraisal of Diagnostic Reliability (QAREL) Checklist for quality appraisal.

Additional outcome(s)
If there is a sufficient number of studies, a meta-analysis may be performed to present the diagnostic accuracy and/or reliability of Node-RADS.

Measures of effect
Measures for diagnostic accuracy will include diagnostic odds ratio, sensitivity and specificity, positive likelihood ratio, negative likelihood ratio, and aera under the receiver operating characteristic curve. Measures for diagnostic reliability will include kappa values, intra-class correlation coefficient, or other suitable statistics.

Data extraction (selection and coding)
A data collection tool will be established based on similar reviews and then trialed on two randomly chosen studies, which fulfilled all the inclusion criteria. These shall be used to train reviewers to appropriately apply the data extraction tool. Two-by-two tables for diagnostic accuracy, and statistics for diagnostic reliability will be directly extracted, if documented, or reconstructed based on available data, for the potential meta-analysis.

Risk of bias (quality) assessment
The studies with diagnostic accuracy results will be assessed using modified Quality Assessment of Diagnostic Accuracy Studies (QUADAS-2) tool for risk of bias and concern on application. The studies with diagnostic reliability results will be assessed using Quality Appraisal of Diagnostic Reliability (QAREL) Checklist for quality appraisal.

Strategy for data synthesis
A narrative synthesis will be provided with information presented in the text and/or tables to summarize and explain the characteristics and findings of the included studies. If a sufficient number of studies, a meta-analysis may be performed to present the diagnostic performance of Node-RADS. The Stata software and/or R language with appropriate packages will be used. The diagnostic odds ratio (DOR) and its corresponding 95% confidence interval (CI) will be quantitatively synthesized as the main effect using the random-effects model, and the corresponding p value will be calculated. Sensitivity, specificity, positive and negative likelihood ratio, and their 95% CIs will be also calculated. A hierarchical summary receiver operating characteristic (HSROC) curve will be plotted to show the diagnostic performance. The category-wise malignancy rates, and inter-observer reliability will be calculated. For heterogeneity assessment, Cochran's Q test and the Higgins I2 test will be used. For publication bias assessment, the Deeks funnel plot will be constructed, and the Deeks funnel asymmetry test will be s performed. Egger's and Begg's tests will be also conducted. A two-tailed p value > 0.10 will be indicate a low publication bias. Sensitivity analysis will be investigated using leave-one-out analysis. The trim and fill method will be employed to evaluate the robustness of meta-analyses.

Analysis of subgroups or subsets
Meta-regression or subgroup analysis will be performed if there is significant heterogeneity among studies. We prospectively selected the following factors for the analysis: region (Western vs non-Western), number of institutions (multicenter vs single center), study design (prospective vs retrospective), subject enrollment (consecutive vs selective), image reviewer (radiologist vs non-radiologist), number of lymph nodes (< median vs ≥ median), reference standard (pathology or composite clinical reference standard vs only pathology), and malignancy rate (< median vs ≥ median).

Type and method of review
Diagnostic; Meta-analysis; Systematic review

Health area of the review
Cancer

Dissemination plans
We plan to present our results of systematic review via presentations on conferences and peer-reviewed journals.

Keywords
Node-RADS; Lymph nodes; Magnetic resonance imaging; Neoplasms; Tomography, X-ray computed

Any additional information
None.

Funding sources/sponsors
This study has received funding by National Natural Science Foundation of China (82302183, 82271934), Yangfan Project of Science and Technology Commission of Shanghai Municipality (22YF1442400), Research Found of Health Commission of Changing District, Shanghai Municipality (2023QN01), Laboratory Open Fund of Key Technology and Materials in Minimally Invasive Spine Surgery (2024JZWC-ZDA03, 2024JZWC-YBA07), and Research Fund of Tongren Hospital, Shanghai Jiao Tong University School of Medicine (TRKYRC-XX202204, TRYJ2021JC06, TRYXJH18, TRYXJH28). They played no role in the study design, data collection or analysis, decision to publish, or manuscript preparation.

Conflicts of interest
None.

Any additional information
This study is supported by TRILOGY, a group of young radiologists from Department of Imaging, Tongren Hospital, Shanghai Jiao Tong University School of Medicine, who work, learn, and play together.

Supplementary Note S2 Search strategy and study selection

1. Search strategy

We firstly performed a preliminary search to confirm the availability of the search string. The search string was developed using the specific terms related to the Node Reporting and Data System 1.0 (Node-RADS) We only included the specific terms of Node-RADS because it is not expectable to identify articles without the term but use this tool. Further, the number of records may be too large to be assessed if we use the term cancer and lymph nodes. We did not use the terms related to imaging modality of CT and MRI. Although the Node-RADS is developed for CT and MRI, we expect to identify studies that expanded the Node-RADS in other imaging procedures, such as CT in PET/CT or MRI in PET/MR.

Then, we conducted the formal search to identify potential available articles. The search string in a previous systematic review (Tian J, Teng F, Xu H, Zhang D, Chi Y, Zhang H. Systematic review and meta-analysis of multiparametric MRI clear cell likelihood scores for classification of small renal masses. Front Oncol. 2022 Oct 26;12:1004502. doi: 10.3389/fonc.2022.1004502. PMID: 36387185; PMCID: PMC9641245.) was also used as a reference. One of the reviewers has experience in developing the search strings. The reviewer developed the search stirrings and validated their feasibility via PubMed. Then the search string was translated for literature search via Embase, Web of Science, China National Knowledge Infrastructure, and Wanfang Data.

The formal literature search and study selection were duplicated by two of three independent reviewers with 6 years of experience in CT and MRI interpretation, with 6 years of experience in medical oncology, and 6 years of experience in general surgery. One of these three reviewers can read articles in English, Chinese, Japanese, German and French; the other one of these three reviewers can read articles in English, Chinese, and Japanese; the last one of these three reviewers can read articles in English, and Chinese. The disagreements were resolved by consults with the review group composed of radiologists, a medical oncologist, a general surgeon, a urologist, a pathologist who was a gynecologist, a dermatologist, an orthopedist, a biostatistical expert, a biomedical engineering expert, a MR scientist, a MR technician, and a journal expert.

1.1 PubMed Search Strategy

Available via https://pubmed.ncbi.nlm.nih.gov
Preliminary search date: 10 Apr 2024
Articles retrieved: 14 (year: 2021-2024)
Formal search date: 15 Apr 2024
Articles retrieved: 14 (year: 2021-2024)

Search string:
"Node Reporting and Data System"[All fields] OR "Node-RADS"[All fields]

1.2 Embase Search Strategy

Available via www.embase.com
Preliminary search date: 10 Apr 2024
Articles retrieved: 17 (year: 2021-2024)
Formal search date: 15 Apr 2024
Articles retrieved: 17 (year: 2021-2024)

Search string:
"Node Reporting and Data System":ti,ab,kw OR "Node-RADS":ti,ab,kw)

1.3 Web of Science Search Strategy

Available via apps.webofknowledge.com
Preliminary search date: 10 Apr 2024
Articles retrieved: 15 (year: 2021-2024)
Formal search date: 15 Apr 2024
Articles retrieved: 15 (year: 2021-2024)

Search string:
TS=("Node Reporting and Data System") OR TS=("Node-RADS")

1.4 China National Knowledge Infrastructure Search Strategy

Available via http://www.cnki.net
Preliminary search date: 10 Apr 2024
Articles retrieved: 0 (year: 2021-2024)
Formal search date: 15 Apr 2024
Articles retrieved: 0 (year: 2021-2024)

Search string:
(TKA="淋巴结报告和数据系统") OR TKA="Node-RADS"

English translation:
("Node Reporting and Data System") OR "Node-RADS"

1.5 Wanfang Data Search Strategy
Available via https://www.wanfangdata.com.cn
Preliminary search date: 10 Apr 2024
Articles retrieved: 0 (year: 2021-2024)
Formal search date: 15 Apr 2024
Articles retrieved: 0 (year: 2021-2024)

Search string:
(主题:("淋巴结报告和数据系统")) OR 主题:("Node-RADS")

English translation:
("Node Reporting and Data System") OR "Node-RADS"

This study search strategy has been tested in a pilot search to confirm its feasibility on 10 Apr 2024. The formal study search was performed on 15 Apr 2024.

2. Study selection
The study selection was performed independently by two of three the same independent reviewers. Contact with the authors was sought if the full-text version was not accessible otherwise. The reference lists of included studies and relevant reviews identified through the search were screened for additional, potentially eligible articles. The disagreements were resolved by consults with the review group composed of radiologists, a medical oncologist, a general surgeon, a urologist, a pathologist who was a gynecologist, a dermatologist, an orthopedist, a biostatistical expert, a biomedical engineering expert, a MR scientist, a MR technician, and a journal expert.

2.1 Study for systematic review
Study inclusion criteria:
1) studies are reported in English, Japanese, Chinese, German or French with institutional full-text availability;
2) the cohort consists of patients with confirmed cancer at any anatomical site;
3) patients had undergone CT or MRI for Node-RADS assessment;
4) with histological results for malignancy lymph nodes, and histological results or composite clinical reference standard for benign lymph nodes.

Study exclusion criteria:
1) duplicate studies;
2) studies published before 2021;
3) reviews, technical reports, letters to editors, comments to published studies, conference proceedings, case reports, brief communications and articles with insufficient information for assessing the methodological quality.

According to the criteria, we assessed the following 12 studies in full-text: 9 studies were included into systematic review, and 3 studies were excluded with justifications.

(1) Included studies for systematic review
1. Gennari AG, Rossi A, Sartoretti T, Maurer A, Skawran S, Treyer V, Sartoretti E, Curioni-Fontecedro A, Schwyzer M, Waelti S, Huellner MW, Messerli M. Characterization of hypermetabolic lymph nodes after SARS-CoV-2 vaccination using PET-CT derived node-RADS, in patients with melanoma. Sci Rep. 2023 Oct 26;13(1):18357. doi: 10.1038/s41598-023-44215-2. PMID: 37884535; PMCID: PMC10603100.
2. Leonardo C, Flammia RS, Lucciola S, Proietti F, Pecoraro M, Bucca B, Licari LC, Borrelli A, Bologna E, Landini N, Del Monte M, Chung BI, Catalano C, Magliocca FM, De Berardinis E, Del Giudice F, Panebianco V. Performance of Node-RADS Scoring System for a Standardized Assessment of Regional Lymph Nodes in Bladder Cancer Patients. Cancers (Basel). 2023 Jan 18;15(3):580. doi: 10.3390/cancers15030580. PMID: 36765540; PMCID: PMC9913205.
3. Leonhardi J, Sabanov A, Schnarkowski B, Hoehn AK, Sucher R, Seehofer D, Denecke T, Meyer HJ. CT Texture Analysis and Node-RADS CT Score of Lymph Nodes in Patients With Perihilar Cholangiocarcinoma. Anticancer Res. 2023 Nov;43(11):5089-5097. doi: 10.21873/anticanres.16709. PMID: 37909955.
4. Loch FN, Beyer K, Kreis ME, Kamphues C, Rayya W, Schineis C, Jahn J, Tronser M, Elsholtz FHJ, Hamm B, Reiter R. Diagnostic performance of Node Reporting and Data System (Node-RADS) for regional lymph node staging of

gastric cancer by CT. Eur Radiol. 2023 Oct 24. doi: 10.1007/s00330-023-10352-5. Epub ahead of print. PMID: 37921924.
5. Lucciola S, Pisciotti ML, Frisenda M, Magliocca F, Gentilucci A, Del Giudice F, Canale V, Scarrone E, Busetto GM, Carrieri G, Cormio L, Carbone A, Pastore A, De Nunzio C, Tubaro A, Leonardo C, Franco G, Di Pierro GB, Salciccia S, Sciarra A, Panebianco V. Predictive role of node-rads score in patients with prostate cancer candidates for radical prostatectomy with extended lymph node dissection: comparative analysis with validated nomograms. Prostate Cancer Prostatic Dis. 2023 Jun;26(2):379-387. doi: 10.1038/s41391-022-00564-z. Epub 2022 Jun 22. PMID: 35732820.
6. Maggialetti N, Greco CN, Lucarelli NM, Morelli C, Cianci V, Sasso S, Rubini D, Scardapane A, Stabile Ianora AA. Applications of new radiological scores: the Node-rads in colon cancer staging. Radiol Med. 2023 Nov;128(11):1287-1295. doi: 10.1007/s11547-023-01703-9. Epub 2023 Sep 14. Erratum in: Radiol Med. 2024 Mar;129(3):524. PMID: 37704777.
7. Meyer HJ, Schnarkowski B, Pappisch J, Kerkhoff T, Wirtz H, Höhn AK, Krämer S, Denecke T, Leonhardi J, Frille A. CT texture analysis and node-RADS CT score of mediastinal lymph nodes - diagnostic performance in lung cancer patients. Cancer Imaging. 2022 Dec 26;22(1):75. doi: 10.1186/s40644-022-00506-x. PMID: 36567339; PMCID: PMC9791752.
8. Yang X, Yang J, Li J, Leng J, Qiu Y, Ma X. Diagnostic Performance of Node Reporting and Data System Magnetic Resonance Imaging Score in Detecting Metastatic Cervical Lymph Nodes of Nasopharyngeal Carcinoma. Clin Med Insights Oncol. 2024 Apr 1;18:11795549241231564. doi: 10.1177/11795549241231564. PMID: 38571681; PMCID: PMC10989040.
9. Wu Q, Lou J, Liu J, Dong L, Wu Q, Wu Y, Yu X, Wang M. Performance of node reporting and data system (node-RADS): a preliminary study in cervical cancer. BMC Med Imaging. 2024 Jan 26;24(1):28. doi: 10.1186/s12880-024-01205-8. PMID: 38279127; PMCID: PMC10811875.

(2) Excluded studies with justifications

1. Elsholtz FHJ, Asbach P, Haas M, Becker M, Beets-Tan RGH, Thoeny HC, Padhani AR, Hamm B. Introducing the Node Reporting and Data System 1.0 (Node-RADS): a concept for standardized assessment of lymph nodes in cancer. Eur Radiol. 2021 Aug;31(8):6116-6124. doi: 10.1007/s00330-020-07572-4. Epub 2021 Feb 14. Erratum in: Eur Radiol. 2021 Mar 19;: PMID: 33585994; PMCID: PMC8270876. **(Node-RADS paper)**
2. Parillo M, van der Molen AJ, Asbach P, Elsholtz FHJ, Laghi A, Ronot M, Wu JS, Mallio CA, Quattrocchi CC. The role of iodinated contrast media in computed tomography structured Reporting and Data Systems (RADS): a narrative review. Quant Imaging Med Surg. 2023 Nov 1;13(11):7621-7631. doi: 10.21037/qims-23-603. Epub 2023 Aug 16. PMID: 37969632; PMCID: PMC10644138. **(Review)**
3. Parillo M, Mallio CA, Van der Molen AJ, Rovira À, Dekkers IA, Karst U, Stroomberg G, Clement O, Gianolio E, Nederveen AJ, Radbruch A, Quattrocchi CC; ESMRMB-GREC Working Group. The role of gadolinium-based contrast agents in magnetic resonance imaging structured reporting and data systems (RADS). MAGMA. 2024 Feb;37(1):15-25. doi: 10.1007/s10334-023-01113-y. Epub 2023 Sep 13. PMID: 37702845; PMCID: PMC10876744. **(Review)**

(3) Extra study identified by browsing the reference lists of included studies and relevant reviews

The browsing of the reference lists of included studies or relevant reviews did not detect extra potentially available study.

2.2 Study for meta-analysis

As predetermined in the review protocol, if there is a sufficient number of studies, a meta-analysis may be performed to present the diagnostic accuracy and/or reliability of Node-RADS. Therefore, we decided to perform the meta-analysis on following questions is conducted: (1) the diagnostic performance, (2) category-wise malignancy rates, and (3) inter-observer reliability of Node-RADS. However, the meta-analysis of inter-observer reliability of Node-RADS was not performed due to the insufficient data. We did not meta-analyze the Node-RADS-MRI and Node-RADS-CT studies, respectively, because the number of the study is limited.

Study inclusion criteria:

(1) studies attempt to answer a similar question.
(2) studies sufficient data to extract the data for meta-analysis, or with those could be calculated using published data.
(3) the studies using largest cohort with histological results, when there were studies using partly or potentially overlapping cohort.

Study exclusion criteria:

(1) insufficient data and unavailable to be calculated after contact to authors.
(2) totally overlapping cohorts.
(3) the property of overlapping cohort cannot be excluded.

According to the criteria, we assessed the following 9 studies in detail, and these 9 studies were included into meta-analysis. There were 9 and 8 studies included for the meta-analysis for the diagnostic performance, and category-wise

malignancy rates, respectively.

The following study was excluded from the meta-analysis for the category-wise malignancy rates, since we cannot extract or reconstruct the data. Loch FN, Beyer K, Kreis ME, Kamphues C, Rayya W, Schineis C, Jahn J, Tronser M, Elsholtz FHJ, Hamm B, Reiter R. Diagnostic performance of Node Reporting and Data System (Node-RADS) for regional lymph node staging of gastric cancer by CT. Eur Radiol. 2023 Oct 24. doi: 10.1007/s00330-023-10352-5. Epub ahead of print. PMID: 37921924.

The pooled inter-observer reliability was not available due to insufficient data.

Supplementary Note S3 Data extraction and quality assessment

1. Data extraction

We developed a data extraction sheet to collect study data. As the reviewers have different levels of experience and knowledge, the items listed were reviewed and discussed to ensure that all reviewers had clear knowledge of the procedures. A training phase was introduced before the formal extraction. During the training phase, two randomly chosen articles from all articles fulfilled the inclusion criteria for discussion were used to train reviewers. They thoroughly read the two randomly chosen articles including the supplementary materials, and measured each study independently. A structured data collection tool was modified and used to help them reach agreement. Disagreements were discussed in order to achieve a shared understanding of each parameter. This pre-defined and piloted data extraction tool was used in the formal data extraction phase. The details of the data extraction tool can be found in Supplementary Table S1.

The data extraction was duplicated by two of three independent reviewers with 6 years of experience in CT and MRI interpretation, with 6 years of experience in medical oncology, and 6 years of experience in general surgery. One of these three reviewers can read articles in English, Chinese, Japanese, German and French; the other one of these three reviewers can read articles in English, Chinese, and Japanese; the last one of these three reviewers can read articles in English, and Chinese. The disagreements were resolved by consults with the review group composed of radiologists, a medical oncologist, a general surgeon, a urologist, a pathologist who was a gynecologist, a dermatologist, an orthopedist, a biostatistical expert, a biomedical engineering expert, a MR scientist, a MR technician, and a journal expert.

The following items has been discussed:

(1) Methodological aspect - Study design

The definition of a retrospective study is clear that retrospectively collect the data and rated Node-RADS for the renal masses. The retrospective analysis of prospective rated Node-RADS is considered as a prospective study in our review. This kind of study prospective rated the Node-RADS as a clinical routine of structured report for renal masses.

(2) Methodological aspect – Node-RADS algorithm

We identified two versions of Node-RADS algorithm. Most of the studies applied the original version of Node-RADS. However, there is one PET/CT study modified the Node-RADS algorithm. We noted this version of Node-RADS as modified Node-RADS.

(3) Diagnostic performance – cutoff for Node-RADS

The Node-RADS document suggested to report Node-RADS 1 and 2 as negative nodes, and Node-RADS 4 and 5 as positive nodes. However, the Node-RADS 3 should be reported depending on the stage and histologic grade of the primary tumor. Therefore, we applied two cutoffs for positive events: Node-RADS ≥ 3 and Node-RADS ≥ 4 for the diagnostic performance analysis. Accordingly, we extracted the two-by-two tables twice for the meta-analysis.

(4) Diagnostic performance - Number of Events (True Positive, False Positive, False Negative, True Negative), Sensitivity, Specificity, Positive Predictive Value (PPV), Negative Predictive Value (NPV), Accuracy

The two-by-two table for each study was directly extracted if reported in the study, or reconstructed with all the available data in the manuscript. These diagnostic performance metrics were extracted for reconstruction of the Number of Events (True Positive, False Positive, False Negative, True Negative) data. If there was an overall diagnostic performance metrics data for the study, we would use the overall data to present the overall diagnostic performance of Node-RADS algorithm by all the observers in the study. However, when overall data was not available, we would use the data form individual observer with the lowest aera of curve or accuracy, to allow conservative estimates for the diagnostic performance of the Node-RADS algorithm.

(5) Category-wise malignancy rates

For the meta-analysis of category-wise malignancy rates, we extracted or reconstructed the number of all assessed node/ patients and the number of positive node/ patients.

(6) Inter-observer reliability

The inter-observer reliability can be assessed by Cohen's kappa, weighted Kappa, etc. The estimate should be reported with 95% conventional interval for the meta-analysis.

2. Quality assessment

2.1 QUADAS-2

The risk of bias assessment of diagnostic performance study was performed using the modified Quality Assessment of Diagnostic Accuracy Studies (QUADAS-2) tool. As the reviewers have different levels of experience and knowledge, the items listed were reviewed and discussed to ensure that all reviewers had clear knowledge of the procedures. A training phase was introduced before the formal assessment. During the training phase, two randomly chosen articles from all articles fulfilled the inclusion criteria for discussion were used to train reviewers. They thoroughly read the two randomly chosen articles including the supplementary materials, and discussed the items in QUADAS-2 tool to reach agreement. The details of the tool can be found in Supplementary Table S2.

The risk of bias assessment of diagnostic performance study was duplicated by two of three independent reviewers with

6 years of experience in CT and MRI interpretation, with 6 years of experience in medical oncology, and 6 years of experience in general surgery. One of these three reviewers can read articles in English, Chinese, Japanese, German and French; the other one of these three reviewers can read articles in English, Chinese, and Japanese; the last one of these three reviewers can read articles in English, and Chinese. The disagreements were resolved by consults with the review group composed of radiologists, a medical oncologist, a general surgeon, a urologist, a pathologist who was a gynecologist, a dermatologist, an orthopedist, a biostatistical expert, a biomedical engineering expert, a MR scientist, a MR technician, and a journal expert.

The following items has been discussed:
(1) Index test - Signaling question 1: were the imaging protocol described in detail?
We discussed how to define a detailed imaging acquisition protocol. We believed that it is not necessary to provide all the imaging parameters for such an image rating system, since it is rated by naked-eye of radiologists. The radiologists can adopt to images with diverse scanners and imaging parameters. Therefore, we define a detailed imaging acquisition protocol as a protocol with all the MRI sequence names for assessment, or all the phase of CT scan for assessment. It is not necessary or possible to force all the institutions that attempt to use the Node-RADS using the same imaging acquisition protocol, but comparable ones.
(2) Index test - Signaling question 2: were the observer trained for Node-RADS before formal rating?
We believe it is necessary to describe the training session of Node-RADS before the formal assessment. The Node-RADS was not a routine content in all the resident or abdominal fellowship training. It is necessary to pre-train the observers before the Node-RADS assessment studies, so that they can use the system properly to present the true diagnostic performance of the system.
(3) Index test - Signaling question 3: was the rating process of Node-RADS approach described in detail (no. of observer, experience, blindness, consensus method)?
We believe it is necessary to describe the rating process of Node-RADS approach in detail, so that the future studies can repeat the study to confirm the results. It is also important to describe the rating process in detail to allow clinical adoption of Node-RADS as a routine in small renal mass assessment. Ideally, the rating process of Node-RADS approach should mimic the clinical reporting process; however, it may be difficult in a “experimental” diagnostic accuracy study. Additionally, the QAREL tool may raise more details for blindness.
(4) Reference - Signaling question 1: was the reference standard adequate (histological evidence from biopsy or surgery)?
All the results of the small renal mass diagnosis should have histological evidence. However, the source of the histological evidence includes surgery and biopsy. It may be not a clinical routine for a mass with low Node-RADS rating to undergo surgery; therefore, we also accept histological evidence from biopsy. However, in comparison to the histological evidence from surgery, it may introduce to some extent risk of bias.
(5) Flow and timing - Signaling question 1: was there an appropriate interval (an interval less than 3 months) between index test and reference standard?
The key of the flow and timing in a diagnostic accuracy study is the interval between the index test and reference standard, i. e., the interval between the imaging and the surgery or biopsy. Not all the studies reported the interval between the imaging and the surgery or biopsy. We considered that these studies should be rated with unclear risk of bias since the interval is unknown. In studies that reported the interval between the imaging and the surgery or biopsy, the key is how to decide whether the interval is adequate. As the follow-up for a renal mass or surgery would be 3, 6, 12, 24… months, which indicated that the mass may change within 3 months. We chose the cutoff of 3 month for the low and high risk of flow and timing assessment.

2.2 QAREL

The risk of bias assessment of inter-observer reliability study was performed using the Quality Appraisal of Diagnostic Reliability (QAREL) tool. As the reviewers have different levels of experience and knowledge, the items listed were reviewed and discussed to ensure that all reviewers had clear knowledge of the procedures. A training phase was introduced before the formal assessment. During the training phase, two randomly chosen articles from all articles fulfilled the inclusion criteria for discussion were used to train reviewers. They thoroughly read the two randomly chosen articles including the supplementary materials, and discussed the items in QAREL tool to reach agreement. The details of the tool can be found in Supplementary Table S3.

The risk of bias assessment of inter-observer reliability study was duplicated by two of three independent reviewers with 6 years of experience in CT and MRI interpretation, with 6 years of experience in medical oncology, and 6 years of experience in general surgery. One of these three reviewers can read articles in English, Chinese, Japanese, German and French; the other one of these three reviewers can read articles in English, Chinese, and Japanese; the last one of these three reviewers can read articles in English, and Chinese. The disagreements were resolved by consults with the review group composed of radiologists, a medical oncologist, a general surgeon, a urologist, a pathologist who was a gynecologist, a dermatologist, an orthopedist, a biostatistical expert, a biomedical engineering expert, a MR scientist, a MR technician, and a journal expert.

The QAREL tool includes 11 items that explore seven principles. Items cover the spectrum of subjects, spectrum of

examiners, examiner blinding, order effects of examination, suitability of the time interval among repeated measurements, appropriate test application and interpretation, and appropriate statistical analysis. The QAREL provides a simple and quick tool for assessing the quality of reliability studies. It has been designed for use in the preparation of systematic reviews, although it may also encourage those involved in diagnostic research to improve the methodology and reporting of reliability studies. QAREL was developed with reference to the items on existing quality appraisal tools and important concepts and items from these existing tools have been incorporated. The reliability of QAREL is dependent on reviewers' understanding of the items on the checklist and also their content knowledge of the studies they are using QAREL to evaluate. Before using QAREL for the evaluation of studies in reviewers of systematic review should form a consensus regarding the appropriate application and interpretation of the test, the suitability of the time interval between each test application, the role of clinical information in the application of the test, and whether there is an accepted reference standard for the condition (or variable) under investigation. Reviewers should also form a consensus about what they consider to be sufficient blinding from clinical information and additional cues not intended to form part of the test. Once the particulars of the study question have been determined and consensus reached, reviewers can proceed to use QAREL to critically appraise the quality of the study.

The following items were considered as "not applicable":
(1) Blindness to prior finding - Were observers blinded to their own prior findings of the test under evaluation?
This item would be rated if observers have examined subjects on more than one occasion. However, in our study, the Node-RADS is only rated once. According to the explanation of this item: If observers only examined subjects on a single occasion, such as with many interobserver reliability studies, reviewers should answer this item as "not applicable". We rated this item as "not applicable" for all studies.
(2) Order of examination - Was the order of examination varied?
This item would be rated if varying the order was important. However, in our study, the Node-RADS all retrospectively rated by the observers. There is not a concept of "order of examination" in our study. According to the explanation of this item: If varying the order of examination was not important, reviewers should select "not applicable". We rated this item as "not applicable" for all studies.

Supplementary Note S4 Data synthesis and analysis

1. Statistical analysis

The statistical analysis was performed with R language version 4.2.1 (https://www.r-project.org/) within RStudio version 1.3.1093 (https://posit.co/) with relevant packages. A two-tailed $p < 0.05$ was recognized as statistical significance, unless specified otherwise. The continuous variables were described as mean ± standard deviation, median (range), while the categorical variables were described as contribution (percentage).

2. Meta-analysis

2.1 Meta-analysis method

The meta- analyses were re-performed with Stata version 15.1 (https://www.stata.com). The data for meta-analysis were extracted directly from the articles or reconstructed using available data. The diagnostic accuracy was estimated with the bivariate random-effects model, while the category-wise malignancy rates and inter-observer reliability were obtained with the random-effects model. The pooled inter-observer reliability was not available due to insufficient data.

The Node-RADS document suggested to report Node-RADS 1 and 2 as negative nodes, and Node-RADS 4 and 5 as positive nodes. However, the Node-RADS 3 should be reported depending on the stage and histologic grade of the primary tumor. Therefore, we applied two cutoffs for positive events: Node-RADS ≥ 3 and Node-RADS ≥ 4 for the diagnostic performance analysis. The two-by-two tables for each cutoff were extracted or reconstructed, respectively. The data of overall diagnostic performance were chosen if reported. Otherwise, the data of the observer with worst diagnostic performance were chosen, to provide a conservative estimate for Node-RADS. The hierarchical summary receiver operating characteristic (HSROC) curve was plotted. The sensitivity, specificity, positive predictive value, negative predictive value, and diagnostic odds ratio were calculated with and their 95% confidence interval (CI) and corresponding *p*-value. The diagnostic odds ratio is selected as the main effect size.

The heterogeneity was estimated by the Cochran's *Q* and the Higgins I^2 statistic. Cochran's *Q* assesses the hypothesis that the distribution of results is homogenous and p-values < 0.05 would generally lead to the rejection of this null-hypothesis. I^2 values of 50% and less are usually considered to be low or unimportant, while above 50% are considered high. We did not conduct *post hoc* subgroup analysis or meta-regression to investigate the potential source of heterogeneity due to the limited number of included studies.

The small study effects were assessed by Egger's test, Begg's test. The publication bias was assessed using Deeks' funnel test and trim and fill method analysis. The 95% prediction interval, and the excess significance bias were conducted. A two-tailed *p*-value < 0.05 was considered as statistically significant, while a two-tailed *p*-value < 0.10 indicated a high risk of publication bias.

2.2 Stata Code for meta-analysis

The following are Stata code used for meta-analysis in this study.

```
// Meta-analysis of diagnostic performance
// The data of author, year, tp = true positive, fp = false positive, fn = false negative, tn = true negative, are prepared
// For sensitivity, specificity, positive likelihood ratio, negative likelihood ratio and diagnostic odds ratio plot
midas tp fp fn tn, res(all)
midas tp fp fn tn, uforest(dss) id (studyid) ford fors
midas tp fp fn tn, uforest(dlr) id (studyid) ford fors
midas tp fp fn tn, texts(0.6) uforest(dlor) id (studyid) ford fors
// For SROC curve plot
midas tp fp fn tn, sroc (both)
// For HSROC curve plot
metandi tp fp fn tn, plot
// For heterogeneity
midas tp fp fn tn, res(het)
// For funnel plot, and Egger's and Begg's test
gen d = sqrt(3) * (log(tp)+log(tn)-log(fp)-log(fn))/3.14
replace d = sqrt(3) * (log(tp+0.5)+log(tn+0.5)-log(fp+0.5)-log(fn+0.5))/3.14 if d==.
gen vard = 3 * (1/tp+1/fp+1/fn+1/tn)/(3.14 * 3.14)
replace vard = 3 * (1/(tp+0.5)+1/(fp+0.5)+1/(fn+0.5)+1/(tn+0.5))/(3.14 * 3.14) if vard==.
gen sed=sqrt(vard)
metafunnel d sed
metabias d sed, egger
metabias d sed, beg
// For Deeks funnel plot, and Deeks funnel plot asymmetry test
midas tp fp fn tn, pubbias
```

```
// For trim and fill method analysis
gen logor = log((tp * tn)/(fp * fn))
replace logor = log(((tp+0.5) * (tn+0.5))/((fp+0.5) * (fn+0.5))) if logor==.
gen selogor = sqrt(1/tp+1/fp+1/fn+1/tn)
replace selogor = sqrt(1/(tp+0.5)+1/(fp+0.5)+1/(fn+0.5)+1/(tn+0.5)) if selogor==.
metatrim logor selogor, eform funnel

// Meta-analysis of category-wise malignancy rates
// The data of of author, year, ass = assigned events, mal = malignant events are prepared
// For the pooled category-wise malignancy rates for each category
metaprop mal ass, random second(fixed) ftt cimethod(extract) label(namevar=studyid)
```

3. Level of evidence

The strength of evidence supporting radiomics for clinical use were categorized into five levels: convincing, highly suggestive, suggestive, weak, and not suggestive. This function is available using the R language version 4.2.1 (https://www.r-project.org/) within RStudio version 1.3.1093 (https://posit.co/) with metaumbralla package via a website App (https://www.metaumbrella.org). The rating was based on the results of a series of aforementioned analyses. The followings are the meta-analyses for determination.

(1) Effect size: Effect size (odds ratio, OR) and the corresponding 95% confidence interval (CI) were pooled as summary effect size using random-effect models and corresponding p values were calculated. This function is available using the metaumbralla package. Because included studies were diagnostic accuracy tests, the diagnostic odds ratio and their 95% CIs were calculated to derive the effect size using a bivariate random effect model.

(2) Heterogeneity: The Cochran's Q test and the I^2 statistic were used to assess heterogeneity among primary studies. Measuring inter-study dispersion assumes that, if all studies were methodologically identical and variation in results were only due to the random selection of study participants, the effect sizes would follow a chi-squared distribution. Cochran's Q assesses the hypothesis that the distribution of results is homogenous and p-values < 0.05 would generally lead to the rejection of this null-hypothesis. As with a small number of studies Cochran's Q can be distorted, I^2, a measure for how much of the variability between effect size estimates is due to methodological heterogeneity rather than sampling error, was also reported. I^2 values of 50% and less are usually considered to be low or unimportant, while above 50% are considered high.

(3) Prediction intervals: The 95% prediction intervals were calculated to facilitate more conservative prediction for potential application of radiomics models (if the number of studies is equal or larger to 3). This function is available using the metaumbralla package. A "notnull" value for the 95% prediction interval of the meta-analysis to exclude the null value to achieve the class for which it is indicated.

(4) Small-study effects: The Egger's test was conducted for small-study effects. When a two-tailed p < 0.10 was reached, small-study effect was considered detected.

(5) Excess significance bias: Excess significance bias was evaluated by a chi-square test comparing the actual observed number of primary studies with a p < 0.05 with the expected number of primary studies with statistical significance. The expected number was the sum of statistical power estimates of each primary study in the meta-analysis. The presence of excess significance bias was proved when observed number > expected number and p < 0.10 for chi-square test were both reached.

The criteria were strongly recommended to be used to all allow an objective, standardized classification of the level of evidence. However, the analysts should not forget that the variables used in these criteria are continuous and the set of cut-off points are only cut-off points. For example, the difference between a factor that includes 1000 patients and a factor that includes 1001 patients is negligible, but according to the criteria, the former can only be class IV (weak), whereas the latter could be class I (convincing).

Supplementary Table S1 Data extraction tool

Field	Item
Bibliographic information	The title of the study
	Published year
	Published journal
	Impact factor of published journal
	Published volume
	Published issue
	Published page
	Study ID, determined by First Author + Year, + A, B, C, if needed
Methodological details	Study design
	Study center
	Study period
	Node-RADS algorithm
	No. of Observer
	Interval between Node-RADS and reference
	Reference standard
Patient characteristics	Inclusion method
	No. of patients
	Age
	Gender
	Cancer type
	Representative sample
	Inclusion criteria
	Exclusion criteria
	No. of node
	Node size
	Histologic diagnosis
Imaging protocol of included studies	Imaging modality
	Standard for protocol
	Scanner
	Imaging protocol
Rating process	No. of observer
	Experience of observer
	Training session
	Representative observer
	Blindness to other observers
	Blindness to prior finding
	Blindness to reference standard
	Blindness to clinical information
	Blindness to additional cues
	Order of examination
	Stability of variable
	Test applicability and interpretation
	Appropriate statistical measures
	Consensus method
	Metrics for agreement assessment (kappa, ICC, etc.)
Diagnostic performance metrics	Number of Events (True Positive)
	Number of Events (False Positive)
	Number of Events (False Negative)
	Number of Events (True Negative)
	Cutoff for Node-RADS
	Sensitivity (SEN)
	Specificity (SPE)
	Accuracy (ACC)
	Positive Predictive Value (PPV)
	Negative Predictive Value (NPV)
	Positive Likelihood Ratio (PLR)
	Negative Likelihood Ratio (NLR)
	Diagnostic Odds Ratio (DOR)

	Aera under curve (AUC)
	Number of malignancy/ assessed node of Node-RADS-1
	Number of malignancy/ assessed node of Node-RADS-2
	Number of malignancy/ assessed node of Node-RADS-3
	Number of malignancy/ assessed node of Node-RADS-4
	Number of malignancy/ assessed node of Node-RADS-5

Supplementary Table S2 QUADAS-2 tool for diagnostic accuracy study with modified questions

Domain and Description	Modified signaling question	Risk of bias	Applicability concern
**1. Patient selection** - describe methods of patient selection: Describe included patients (prior testing, presentation, intended use of index test and setting)	Signaling question 1: was the type of study (retrospective or prospective) specified?	Could the selection of patients have introduced bias?	Are there concerns that the included patients do not match the review question?
	Signaling question 2: were the study institution and inclusion period clearly presented?		
	Signaling question 3: were the inclusion/exclusion criteria specified?		
**2. Index test** - describe the index test and how it was conducted and interpreted	Signaling question 1: were the imaging protocol described in detail?	Could the conduct or interpretation of the index test have introduced bias?	Are there concerns that the index test, its conduct, or interpretation differ from the review question?
	Signaling question 2: were the observer trained for Node-RADS before formal rating?		
	Signaling question 3: was the rating process of Node-RADS approach described in detail (no. of observer, experience, blindness issues, consensus method)?		
**3. Reference standard** - describe the reference standard and how it was conducted and interpreted	Signaling question 1: was the reference standard adequate (histological evidence from biopsy or surgery)?	Could the reference standard, its conduct, or its interpretation have introduced bias?	Are there concerns that the target condition as defined by the reference standard does not match the review question?
	Signaling question 2: was the reference standard established by an experienced pathologist with an adequate cirteria?		
**4. Flow and timing** - describe any patients who did not receive the index test(s) and/or reference standard or who were excluded from the 2x2 table (refer to flow diagram): Describe the time interval and any interventions between index test(s) and reference standard	Signaling question 1: was there an appropriate interval (an interval less than 3 months) between index test and reference standard?	Could the patient flow have introduced bias?	n. a.

Note: Whiting PF, Rutjes AW, Westwood ME, Mallett S, Deeks JJ, Reitsma JB, Leeflang MM, Sterne JA, Bossuyt PM; QUADAS-2 Group. QUADAS-2: a revised tool for the quality assessment of diagnostic accuracy studies. Ann Intern Med. 2011 Oct 18;155(8):529-36. doi: 10.7326/0003-4819-155-8-201110180-00009. PMID: 22007046.

Supplementary Table S3 QAREL tool for diagnostic reliability study with consensus on items

Item	Method for rating this item
**1. Representative sample** - Was the test evaluated in a sample of subjects who were representative of those to whom the authors intended the results to be applied?	(1) What was the study question, and to whom did the authors intend the results to be applied?<br>(2) What were the characteristics of the study sample (e.g., age, weight, height, symptom status, and diagnosis)? This is important to help identify potential selection bias and to determine to whom the results are applicable.<br>(3) In what setting was the study conducted (e.g., tertiary care, primary care, university or educational setting)? This is important to help identify potential spectrum and selection bias and to determine to whom the results are applicable.<br>(4) How were subjects recruited for the study? Were sub- jects selected by convenience sampling, were they consecutive, or randomly sampled? This is important to help identify selection bias.<br>(5) Were selection criteria applied? The applicability of a study is affected by the selection of subjects. Sub- jects who represent typical patients on whom the test would be used in practice should be used in the study of clinical tests. For instance, it is inappropriate to use normal subjects to establish the reliability of clinical measures. It is appropriate, however, to use normal or healthy volunteers if the aim of the test is to detect normative data, for example, the reliability of physio- therapists to identify the spinous processes of lumbar vertebrae.<br>(6) Were there any dropouts or missing data? If data are missing, this may create a biased sample that is not representative of those to whom the results were in- tended to apply or those who would normally receive the test in practice.<br>Firstly, establish the study question and the population of interest to the investigators. If subjects were representative of those to whom the authors intended the results to be applied, select “yes” If the subjects were not representative, select “no” and if unsure or if insufficient information was provided, select “unclear”
**2. Representative rater** - Was the test performed by raters who were representative of those to whom the authors intended the results to be applied?	(1) What was the study question, and to whom did the authors intend the results to be applied?<br>(2) What were the characteristics of the raters? This relates to the raters’ qualifications, specialization, their experience in performing the test under investigation, and whether they received any specific training for the study.<br>(3) In what setting was the study conducted? (e.g., tertiary care, primary care, university or educational set- ting) This is important to help determine the type of raters the results are applicable to.<br>(4) Were selection criteria applied? This is important to help determine the type of raters the results are applicable to.<br>(5) Were there any dropouts or missing data? If data are missing this may have the effect of creating a biased sample of raters.<br>If the test was per- formed by raters who were representative of those to whom the authors intended the results to be applied, select “yes” If the raters were not representative, select “no” If unsure or if insufficient information was provided, select “unclear”
**3. Blindness to other rater** - Were raters blinded to the findings of other raters during the study?	If two or more raters were included in the study, reviewers should determine if they were blinded to each other’s’ findings and select either “yes” or “no”. Reviewers should select “unclear” if they are unsure or if insufficient information was provided. If only one rater was included, reviewers should select “not applicable”.
**4. Blindness to prior finding** - Were raters blinded to their own prior findings of the test under evaluation?	If raters have examined subjects on more than one occasion, reviewers should choose either “yes” if raters were blinded to subjects, they had examined previously, or “no” if raters were not blinded. Reviewers should select “unclear” if they are unsure or if insufficient information was provided. If raters only examined subjects on a single occasion, such as with many interrater reliability studies, reviewers should answer this item as “not applicable”
**5. Blindness to reference standard** - Were raters blinded to the subjects’ disease status or the results of the accepted reference standard for the target dis- order (or variable) being evaluated?	Reviewers should select “not applicable” in those instances in which no accepted reference standard is available or if the subjects have not been evaluated with the reference standard. If the true dis- ease status or result of a reference standard for subjects is known, then reviewers would select from either “yes” if raters were blinded to the results of the reference standard, or “no” if raters were not blinded to the results of the reference standard. Reviewers should select “unclear” if they are unsure or if insufficient information was provided.

Eur Radiol (2024) Zhong JY, Mao SQ, Chen HD, et al.

**6. Blindness to clinical information** - Were raters blinded to clinical information that was not intended to form part of the study design or testing procedure?	Answer "yes" if raters were blinded to clinical information that was not intended to form part of the test and "no" if raters were not blinded. Reviewers should select "unclear" if they are unsure or if insufficient information was provided.
**7. Blindness to additional cues** - Were raters blinded to additional cues that are not part of the test?	Answer "yes" if there is a clear description about how raters were blinded to additional cues or "no" if it is clear that they were not blinded. Reviewers should select "unclear" if they are unsure or if there is insufficient information provided with which to assess this item.
**8. Order of examination** - Was the order of examination varied?	If varying the order was important, reviewers should select "yes" if the order was varied and "no" if the order was not varied. Reviewers should select "unclear" if they are unsure if the order was varied or if the article does not provide sufficient information. If varying the order of examination was not important, reviewers should select "not applicable"
**9. Stability of variable** - Was the stability (or theoretical stability) of the variable being measured taken into account when determining the suitability of the time interval among repeated measures?	Select "yes" if the time interval was appropriate or "no" if the time interval was not appropriate. Reviewers should select "unclear" if they are unsure or if insufficient information was provided.
**10. Test applicability and interpretation** - Was the test applied correctly and interpreted appropriately?	(1) Did the researchers apply the test correctly? The use of the word "correctly" is used to mean that the test was performed according to current standards or was applied in accordance with the aims and objectives of the study. The incorrect application of a test can in- validate an otherwise good quality study. Studies that report new tests or modifications of existing tests should include a detailed description of the test, including the rationale, procedure, and interpretation. A change in test procedure may have a substantial im- pact on the performance of the test. Although this may not affect the quality of the study, it does affect the study question and applicability of the results.<br>(2) If the test results require interpretation, were the criteria for the interpretation appropriate? If the criteria for the interpretation of the test are incorrect or irregular, then this may have an impact on the quality of the study. For example, if two or more examiners ap- ply a different threshold for determining what is nor- mal or abnormal, this would introduce measurement bias and reduce the estimates of reliability.<br>Reviewers should select "yes" if the test was applied correctly and if the results were interpreted appropriately. Select "no" if either the test was applied incorrectly or if the results were not interpreted appropriately. Reviewers should select "unclear" if they are unsure or if insufficient information was provided.
**11. Appropriate statistical measures** - Were appropriate statistical measures of agreement used?	Reviewers should select "yes" if the analysis was appropriate and "no" if the analysis was not appropriate. Reviewers should select "unclear" if they are unsure or if insufficient information was provided.

Note: Lucas NP, Macaskill P, Irwig L, Bogduk N. The development of a quality appraisal tool for studies of diagnostic reliability (QAREL). J Clin Epidemiol. 2010 Aug;63(8):854-61. doi: 10.1016/j.jclinepi.2009.10.002. Epub 2010 Jan 13. PMID: 20056381.

Supplementary Table S4 Category of five levels of evidence based on meta-analyzes

**Levels of supporting evidence**	**Description**
Convincing	$p < 10^{-6}$, > 1000 events, the largest study reaches statistical significance ($p < 0.05$), I2 < 50%, the null value excluded by the 95% PI, no small-study effects ($p > 0.1$ for Egger's test) and excess significance ($p > 0.1$).
Highly suggestive	$p < 10^{-6}$, > 1000 events, the largest study reaches statistical significance ($p < 0.05$)
Suggestive	$p < 10^{-3}$, > 1000 events
Weak	$p < 0.05$
Not suggestive	$p > 0.05$

Note: Fusar-Poli P, Radua J. Ten simple rules for conducting umbrella reviews. Evid Based Ment Health. 2018 Aug;21(3):95-100. doi: 10.1136/ebmental-2018-300014. Epub 2018 Jul 13. PMID: 30006442.

Supplementary Table S5 Methodological aspect of included studies

Study	Author	Year	Journal	Study design	Study center	Study period	Cancer type	Imaging modality	No. of Observer	Interval between Node-RADS and reference	Reference standard
Gennari 2023 [37]	Gennari	2023	Sci Rep	Retrospective	University Hospital of Zurich	2021.02 – 2021.04	Melanoma	CT	1	NR	CRRS
Leonardo2023 [38]	Leonardo	2023	Cancers (Basel)	Retrospective	Sapienza University of Rome	2019.01 – 2022.06	Bladder cancer	CT	1	NR	Histology
Leonhardi2023 [39]	Leonhardi	2023	Anticancer Res	Retrospective	University of Leipzig	2016.01 – 2021.12	Cholangiocarcinoma	CT	2	< 1 month	Histology
Loch2023 [40]	Loch	2023	Eur Radiol	Retrospective	Charité – Universitätsmedizin Berlin	2016.01 – 2022.06	Gastric cancer	CT	2	NR	Histology
Lucciola 2023 [41]	Lucciola	2023	Prostate Cancer Prostatic Dis	Retrospective	University Sapienza of Rome	2018.01 – 2021.12	Prostate cancer	MRI	1	NR	Histology
Maggialetti2023 [42]	Maggialetti	2023	Radiol Med	Retrospective	Polyclinic of Bari	2021.04 – 2022.05	Colon cancer	CT	2	< 2 months	Histology
Mayer2022 [43]	Mayer	2022	Cancer Imaging	Retrospective	Leipzig University	2012.01 – 2015.12	Lung cancer	CT	2	< 1 month	Histology
Wu2024 [44]	Wu	2024	BMC Med Imaging	Retrospective	Henan Provincial People's Hospital	2017.03 – 2022.02	Cervical cancer	MRI	2	NR	Histology
Yang2024 [45]	Yang	2024	Clin Med Insights Oncol	Retrospective	West China Hospital of Sichuan University	2012.07 – 2022.09	Nasopharyngeal Carcinoma	MRI	2	NR	Histology

CCRS = composite clinical reference standard

Supplementary Table S6 Patient characteristics of included studies

Study	Patient inclusion	Cancer type	No. of patient	Age	Gender (M/F)	Inclusion criteria	Exclusion criteria
Gennari2023 [37]	selective	Melanoma	54 vaccinated; 54 unvaccinated	68.7 ± 13.9; 66.6 ± 14.5	39/15; 37/17	(1) age > 18 years old; (2) diagnosis of melanoma; (3) having undergone a 2-[18F]-FDG PET/CT	(1) inadequate quality of low-dose CT images; (2) previous metastatic involvement of axillary LNs, defined as HALNs detected in the penultimate 2-[18F]-FDG PET/CT exam; (3) absence of subcutaneous tissue in the axillary or pectoral region; (4) presence of concomitant tumors.
Leonardo2023 [38]	consecutive	Bladder cancer	49	70 (61, 77)	37/12	patients with a diagnosis of either muscle-invasive bladder cancer or high-risk non- muscle-invasive bladder cancer, unresponsive to intravesical therapies, treated with RC plus bilateral pelvic lymph node dissection	(1) preoperative systemic chemotherapy; (2) patients for whom a preoperative CT scan; (3) patients who did not receive an extended pelvic lymph node dissection; (4) patients who did not have a packeted lymph node submission; (5) patients with missing data
Leonhardi2023 [39]	consecutive	Cholangiocarcinoma	25	72.4 ± 8.1	16/9	Available presurgical CT images of histopathologically confirmed perihilar cholangiocarcinoma	NR
Loch2023 [40]	consecutive	Gastric cancer	91	66 (33-91)	54/37	patients with histologically proven gastric adenocarcinoma who underwent surgery at the Department of Surgery, Cam- pus Benjamin Franklin, Charité – Universitätsmedizin Berlin, Germany	(1) Entity other than adenocarcinoma; (2) Emergency surgery with accompanied peritonitis; (3) Priorly performed partial gastrectomy in patients history; (4) Neoadjuvant radio-chemotherapy (5) EBV-associated carcinoma; (6) No preoperative abdominal CT, CT > 50 days prior to surgery or insufficient quality; (7) Palliative gastrectomy in metastatic disease
Lucciola2023 [41]	consecutive	Prostate cancer	150	65.9 ± 6.3	150/0	(1) histological diagnosis of prostatic adenocarcinoma; no distant metastases at clinical staging; (2) high risk or intermediate risk disease with an estimated risk for pN+ higher than 5% using the	(1) androgen deprivation therapies; (2) chemotherapies, pelvic radiation therapies or treatments with other agents that could influence prostate tumor growth and diffusion

						Briganti or 7% using the Gandaglia nomogram incorporating mpMR-targeted biopsies;<br>(3) radical prostatectomy as chosen primary treatment decision after multidisciplinary discussion of treatment options and presentation to the patient;<br>(4) anatomical extended lymph node dissection with removal of the obturator, internal iliac, external iliac lymph nodes associated to radical prostatectomy	
Maggialetti2023 [42]	consecutive	Colon cancer	67	69.4 ± 9.0	47/20	(1) a preoperative abdominal CT scan with contrast;<br>(2) histological diagnosis of colon cancer;<br>(3) patients who underwent excisional surgery within 2 months from diagnosis with LN histological results;<br>(4) participants who agreed to informed consent for abdominal CT scan with contrast	(1) patients who had only a postoperative abdominal CT scan;<br>(2) patients who did not had a non-contrast abdominal CT scan;<br>(3) did not have previous TC imaging (such as only MRI images);<br>(4) no histological confirmation of the tumor;<br>(5) patients with prior preoperative therapy.
Mayer2022 [43]	consecutive	Lung cancer	91	64.8 ± 10.8	59/32	(1) sufficient presurgical or prebiopsy CT images;<br>(2) histopathologically confirmed primary lung cancer;<br>(3) histopathological mediastinal lymph node analysis.	NR
Wu2024 [44]	consecutive	Cervical cancer	81	49.8 ± 10.6	0/81	(1) patients who underwent radical hysterectomy and lymph node dissection;<br>(2) patients who underwent pelvic MRI with gadolinium contrast for preoperative evaluation;<br>(3) the time interval between pelvic MRI and surgery was within 7 days;<br>(4) the pathological status of lymph nodes were evaluated according to regions	(1) patients underwent therapy including neoadjuvant chemotherapy or conization before surgery;<br>(2) the pathological evaluation of lymph node status was not detailed according to regions.

Yang2024 [45]	consecutive	Nasopharyngeal Carcinoma	119	47.6	85/34	(1) a pathological diagnosis of NPC CLN metastasis were enrolled as the positive group;<br>(2) lymph node biopsy or dissection with pathologic confirmation of benign nodes as the negative group	(1) patients without available MRI scans;<br>(2) those with unavailable data

Supplementary Table S7 Imaging protocol of included studies

Study	Imaging modality	Standard for protocol	Scanner	Imaging protocol
Gennari2023 [37]	CT	NR	PET/CT scanner (GE Discovery MI, GE Healthcare, Waukesha, WI)	A low-dose, attenuation correction, spiral CT scan (collimation width: 0.625 mm, pitch: 0.98, kVp: 120, automatic tube dose modulation ranging between 15 and 100 mAs, matrix: 512 × 512, slice thickness: 1.25 mm, spacing between slices: 1.25 mm), displayed using body filter (W/L: 40/400 Hounsfield Unit, HU)
Leonardo2023 [38]	CT	Protocol recommended by the manufacturer	Somatom Sensation 128, Somatom Sensation 64 (Siemens Healthcare, Victoria, Australia)	The baseline, arterial, nephrographic, and excretory phases of the abdomen and pelvis were acquired for the evaluation of the entire excretory tract. Multiplanar (axial, coronal, and sagittal) images were reconstructed with a slice thickness of 1 mm, using the classical filtered back- projection method with a soft tissue kernel of B20 and a bone kernel of B60.
Leonhardi2023 [39]	CT	NR	128-slice CT scanner (Ingenuity 128, Philips, Hamburg, Germany)	For every patient, intravenous administration of an iodine-based contrast medium (90 ml Imeron 400 MCT, Bracco, Imaging Germany GmbH, Konstanz, Germany) was given at a rate of 2-4.0 ml/s *via* a peripheral venous line. All investigated CT images were obtained in portal venous phase after 70 s. Automatic bolus tracking was performed in the aorta descenders with a trigger of 100 Hounsfield units (HU). Typical imaging parameters were: 100 kVp; 125 mAs; slice thickness,1 mm; pitch, 0.9.
Loch2023 [40]	CT	NR	NR	Minimum quality was defined as contrast-enhanced CT with a slice thickness of at least 5 mm and all CT images were assessed.
Lucciola2023 [41]	MRI	NR	NR	Multiple sequence MR scan.
Maggialetti2023 [42]	CT	structured CT protocol proposed by Granata V, Faggioni L. et al. in “Structured reporting of computed tomography in the staging of colon cancer: a Delphi consensus proposal”	Siemens Somatom Definition DS	Images were displayed non-contrast phase, venous phase and delayed phase, reconstructed in multiplanar (axial, coronal and sagittal) images using multiplanar reformatting (MPR) for the best assessment of nodes’ short axis and characteristics.
Mayer2022 [43]	CT	NR	a 128-slice CT scanner (Inge- nuity 128, Philips, Hamburg, Germany)	In 42% of patients, intravenous iodine-based contrast medium (60mL Imeron 400 MCT, Bracco Imaging Ger- many GmbH, Konstanz, Germany) was injected at a rate of 4.0mL/s via a peripheral venous line. Automatic bolus tracking was performed in the aorta descenders with a trigger of 100 Hounsfield units (HU). Typical imaging parameters were: 100 kVp; 125 mAs; slice thick- ness,1 mm; pitch, 0.9.
Wu2024 [44]	MRI	NR	Discovery MR 750 (GE Medical Systems, Milwaukee, WI, USA) or TrioTim (Siemens	A table for MRI protocol is provided with following parameters, Scanne, Sequence TR (ms), TE (ms), Slice Thickness (mm), Slice Gap (mm), Bandwidth, Slices.

			Healthcare)	
Yang2024 [45]	MRI	NR	3.0T scanner (Philips, Eindhoven, the Netherlands)	Head and neck T1- and T2-weighted MRI images (T1WI and T2WI) were acquired with a slice thickness of 3mm (T1WI echo time (TE)/repetition time (TR)/inversion time (TI)=2.950/6.600/0.000ms; T2WI TE/TR/TI=95.360/4078.000/0.000ms). The manufacturer's recommended acquisition parameters were used.

Supplementary Table S8 Rating process of included studies

Study	No. of observer	Experience of observer	Training session and assessment approach	Node-RADS	Blindness to other observers	Blindness to prior finding	Blindness to reference standard	Blindness to clinical information	Blindness to additional cues	Consensus method	Statistical measures
Gennari2023 [37]	1	7 years of experience in oncologic imaging	A four-step approach	Adapted Node-RADS classification	N. a.	N. a.	Unclear	Yes	Yes	N. a.	N. a.
Leonardo2023 [38]	1	15 years of experience in the field of CT imaging	Guiding by a Node-RADS three-level flowchart	Original Node-RADS	N. a.	N. a.	Yes	Unclear	Unclear	N. a.	N. a.
Leonhardi2023 [39]	2	2 and 4 years of experience in oncological CT imaging	According to Node-RADS score	Original Node-RADS	Yes	N. a.	Yes	Unclear	Unclear	Unclear	Cohen's kappa
Loch2023 [40]	2	over 10 years of experience in abdominal cross-sectional imaging; 2 years of experience	According to Node-RADS score	Original Node-RADS	Yes	N. a.	Yes	Unclear	Unclear	Unclear	Cohen's kappa
Lucciola2023 [41]	1	15 years of experience in MR imaging	Guiding by a Node-RADS three-level flowchart	Original Node-RADS	N. a.	N. a.	Yes	Unclear	Unclear	N. a.	N. a.
Maggialetti2023 [42]	2	25 and 17 years of experience	Guiding by a Node-RADS	Original Node-RADS	Yes	N. a.	Yes	Unclear	Unclear	Unclear	NR

			three-level flowchart									
Mayer2022 [43]	2	3 and 2 years of experience in CT imaging analysis	According to Node-RADS score	Original Node-RADS	Unclear	N. a.	Unclear	Unclear	Unclear	Unclear	Cohen’s kappa	
Wu2024 [44]	2	13 and 12 years of experience in pelvic imaging	According to Node-RADS score	Original Node-RADS	Yes	N. a.	Yes	Unclear	Unclear	Unclear	Weighted Kappa	
Yang2024 [45]	2	8 and 5 years of experience in MR imaging	According to Node-RADS score	Original Node-RADS	Unclear	N. a.	Unclear	Unclear	Unclear	Unclear	Weighted Kappa	

Supplementary Table S9 QUADAS-2 assessment by two reviewers and consensus results

Study	Risk of bias				Applicability concern		
	Patient selection	Index test	Reference standard	Flow and timing	Patient selection	Index test	Reference standard
Gennari2023 [37]	L/L/L	H/H/H	H/U/H	U/U/U	L/L/L	U/L/U	Unclear
Leonardo2023 [38]	L/L/L	L/L/L	L/L/L	U/U/U	L/L/L	L/L/L	L/L/L
Leonhardi2023 [39]	U/L/U	H/H/H	L/L/L	L/L/L	L/L/L	U/L/U	L/L/L
Loch2023 [40]	L/L/L	H/H/H	L/L/L	U/U/U	L/L/L	U/L/U	L/L/L
Lucciola2023 [41]	L/L/L	H/H/H	L/L/L	U/U/U	L/L/L	U/L/U	L/L/L
Maggialetti2023 [42]	L/L/L	L/L/L	L/L/L	L/L/L	L/L/L	L/L/L	L/L/L
Mayer2022 [43]	U/L/U	H/H/H	L/L/L	L/L/L	L/L/L	U/L/U	L/L/L
Wu2024 [44]	L/L/L	H/H/H	L/L/L	U/U/U	L/L/L	U/L/U	L/L/L
Yang2024 [45]	L/L/L	H/H/H	L/L/L	U/U/U	L/L/L	U/L/U	L/L/L

Note: Whiting PF, Rutjes AW, Westwood ME, Mallett S, Deeks JJ, Reitsma JB, Leeflang MM, Sterne JA, Bossuyt PM; QUADAS-2 Group. QUADAS-2: a revised tool for the quality assessment of diagnostic accuracy studies. Ann Intern Med. 2011 Oct 18;155(8):529-36. doi: 10.7326/0003-4819-155-8-201110180-00009. PMID: 22007046.
L = Low risk, H = High risk, U = Unclear risk. The results of assessment are presented in the first reviewer/ the second reviewer/ the final results with consensus.

Supplementary Table S10 QAREL assessment by two reviewers and consensus results

Study	Representative sample	Representative rater	Blindness to other raters	Blindness to prior finding	Blindness to reference standard	Blindness to clinical information	Blindness to additional cues	Order of examination	Stability of variable	Test applicability and interpretation	Appropriate statistical measures
Gennari2023 [37]	N. a.	N. a.	N. a.	N. a.	N. a.	N. a.	N. a.	N. a.	N. a.	N. a.	N. a.
Leonardo2023 [38]	N. a.	N. a.	N. a.	N. a.	N. a.	N. a.	N. a.	N. a.	N. a.	N. a.	N. a.
Leonhardi2023 [39]	Y/Y/Y	Y/Y/Y	Y/Y/Y	N. a.	Y/Y/Y	U/N/U	U/N/U	N. a.	U/N/U	Y/Y/Y	Y/Y/Y
Loch2023 [40]	Y/Y/Y	Y/Y/Y	Y/Y/Y	N. a.	Y/Y/Y	U/N/U	U/N/U	N. a.	U/N/U	Y/Y/Y	Y/Y/Y
Lucciola2023 [41]	N. a.	N. a.	N. a.	N. a.	N. a.	N. a.	N. a.	N. a.	N. a.	N. a.	N. a.
Maggialetti2023 [42]	Y/Y/Y	Y/Y/Y	Y/Y/Y	N. a.	Y/Y/Y	U/N/U	U/N/U	N. a.	U/N/U	Y/Y/Y	U/U/U
Mayer2022 [43]	Y/Y/Y	Y/Y/Y	U/Y/U	N. a.	U/N/U	U/N/U	U/N/U	N. a.	U/N/U	Y/Y/Y	Y/Y/Y
Wu2024 [44]	Y/Y/Y	Y/Y/Y	Y/Y/Y	N. a.	Y/Y/Y	U/N/U	U/N/U	N. a.	U/N/U	Y/Y/Y	Y/Y/Y
Yang2024 [45]	Y/Y/Y	Y/Y/Y	U/Y/U	N. a.	U/N/U	U/N/U	U/N/U	N. a.	Y/Y/Y	Y/Y/Y	Y/Y/Y

Note: Lucas NP, Macaskill P, Irwig L, Bogduk N. The development of a quality appraisal tool for studies of diagnostic reliability (QAREL). J Clin Epidemiol. 2010 Aug;63(8):854-61. doi: 10.1016/j.jclinepi.2009.10.002. Epub 2010 Jan 13. PMID: 20056381.

Y = Yes, N = No, U = Unclear risk, N. a. = not applicable. The results of assessment are presented in the first reviewer/ the second reviewer/ the final results with consensus.

Supplementary Table S11 Two-by-two data for meta-analysis

Study	Level	No. of assigned	No. of malignancies	TP	FP	FN	TN	SEN	SPE	PPV	NPV	ACC
Node-RADS ≥ 3 as positive												
Gennari2023 [37]	Node	216	14	14	28	0	174	100.0%	86.1%	33.3%	100.0%	87.0%
Leonardo2023 [38]	Patient	49	14	11	8	3	27	78.6%	77.1%	57.9%	90.0%	77.6%
Leonhardi2023 [39]	Node	50	21	18	2	3	27	85.7%	93.1%	90.0%	90.0%	90.0%
Loch2023 [40]	Patient	91	37	21	5	16	49	56.8%	90.7%	80.8%	75.4%	76.9%
Lucciola2023 [41]	Patient	150	36	8	5	28	109	22.2%	95.5%	61.5%	79.3%	77.7%
Maggialetti2023 [42]	Patient	67	28	26	3	2	36	92.9%	92.3%	89.7%	94.7%	92.5%
Mayer2022 [43]	Patient	91	35	22	3	13	53	62.9%	94.6%	88.0%	80.3%	82.4%
Wu2024 [44]	Patient	81	40	27	7	13	34	67.5%	82.9%	79.4%	72.3%	75.3%
Yang2024 [45]	Node	203	140	129	8	11	55	92.1%	87.3%	94.2%	83.3%	90.6%
Node-RADS ≥ 4 as positive												
Gennari2023 [37]	Node	216	14	8	5	6	197	57.1%	97.5%	61.5%	97.0%	94.9%
Leonardo2023 [38]	Patient	49	14	8	5	6	30	57.1%	85.7%	61.5%	83.3%	77.6%
Leonhardi2023 [39]	Node	50	21	14	0	7	29	66.7%	100.0%	100.0%	80.6%	86.0%
Loch2023 [40]	Patient	91	37	18	1	19	53	48.6%	98.1%	94.7%	73.6%	78.0%
Lucciola2023 [41]	Patient	150	36	6	0	30	114	16.7%	100.0%	100.0%	79.2%	80.0%
Maggialetti2023 [42]	Patient	67	28	18	1	10	38	64.3%	97.4%	94.7%	79.2%	83.6%
Mayer2022 [43]	Patient	91	35	14	0	21	56	40.0%	100.0%	100.0%	72.7%	76.9%
Wu2024 [44]	Patient	81	40	20	2	20	39	50.0%	95.1%	90.9%	66.1%	72.8%
Yang2024 [45]	Node	203	140	109	2	31	61	77.9%	96.8%	98.2%	66.3%	83.7%

Supplementary Table S12 Category-wise malignancy rate for meta-analysis

Study	Level	Node-RADS-1		Node-RADS-2		Node-RADS-3		Node-RADS-4		Node-RADS-5	
		Mal	Ass	Mal	Ass	Mal	Ass	Mal	Ass	Mal	Ass
Gennari2023 [37]	Node	0	152	0	22	6	29	7	12	1	1
Leonardo2023 [38]	Patient	0	20	3	10	3	6	3	7	5	6
Leonhardi2023 [39]	Node	0	18	3	12	4	6	10	10	4	4
Loch2023 [40]	Patient	NR	31	NR	33	NR	7	NR	12	NR	7
Lucciola2023 [41]	Patient	4	109	24	26	2	7	6	6	0	0
Maggialetti2023 [42]	Patient	1	28	1	10	8	10	10	11	8	8
Mayer2022 [43]	Patient	4	45	9	21	8	11	6	6	8	8
Wu2024 [44]	Patient	6	23	7	24	3	7	4	5	20	22
Yang2024 [45]	Node	5	47	6	19	20	26	45	47	64	64

Note: Mal = number of malignancy, Ass = number of assigned

Supplementary Table S13 Inter-observer reliability for meta-analysis

Study	No. of observers	Metrics	Measure	95% CI	Interpretation
Gennari2023 [37]	1	N. a.	N. a.	N. a.	N. a.
Leonardo2023 [38]	1	N. a.	N. a.	N. a.	N. a.
Leonhardi2023 [39]	2	Cohen's kappa	0.35	NR	Fair
Loch2023 [40]	2	Cohen's kappa	0.67	NR	Moderate
Lucciola2023 [41]	1	N. a.	N. a.	N. a.	N. a.
Maggialetti2023 [42]	2	NR	NR	NR	NR
Mayer2022 [43]	2	Cohen's kappa	0.48	NR	Moderate
Wu2024 [44]	2	Weighted Kappa	0.829, 0.593, 0.547, and 0.556	NR	Fair to Substantial
Yang2024 [45]	2	Weighted Kappa	0.863	0.830-0.897	Substantial

Supplementary Figure S1 Meta-analysis of diagnostic performance for Node-RADS ≥ 3 as positive

(A) Forest plot of sensitivity and (B) Forest plot of specificity

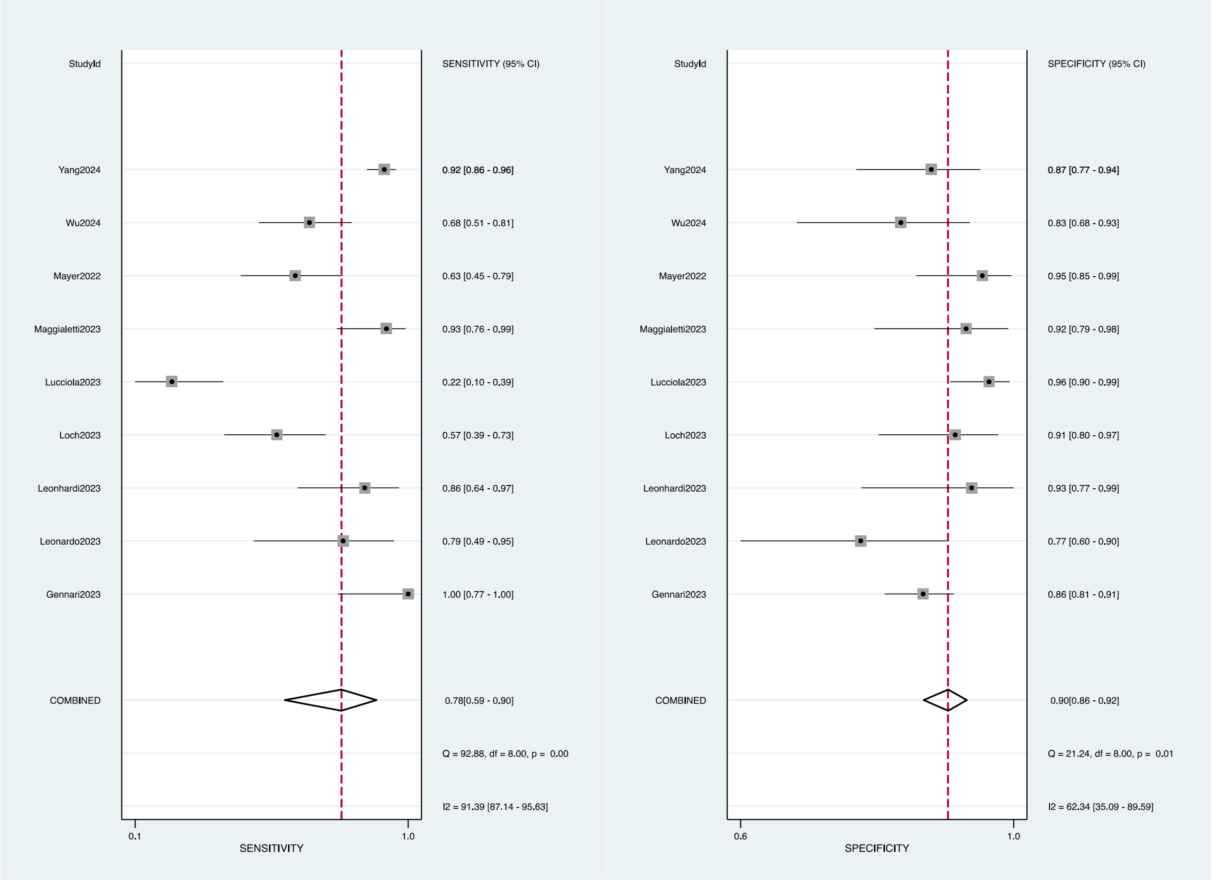

(C) Forest plot of positive likelihood ratio and (D) Forest plot of negative likelihood ratio

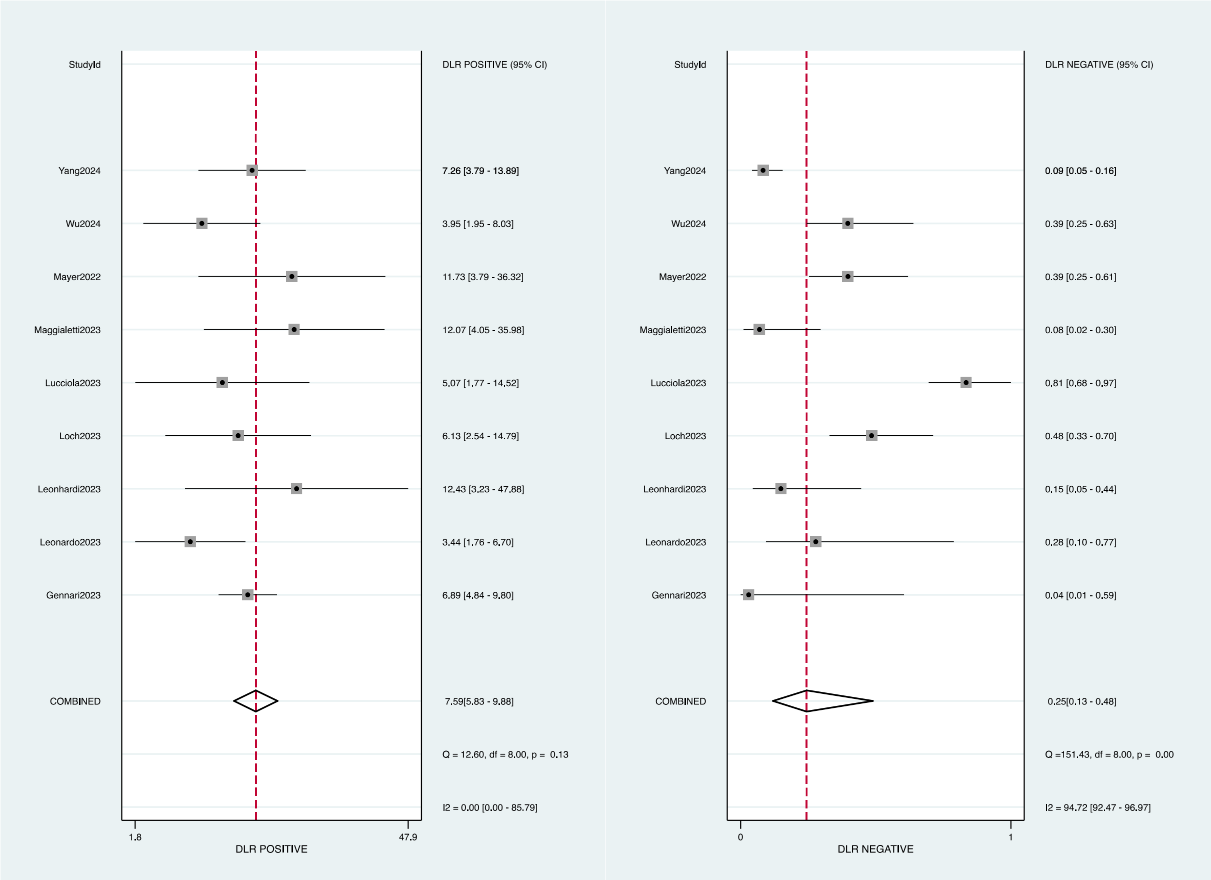

(E) Summary receiver operating characteristic curve

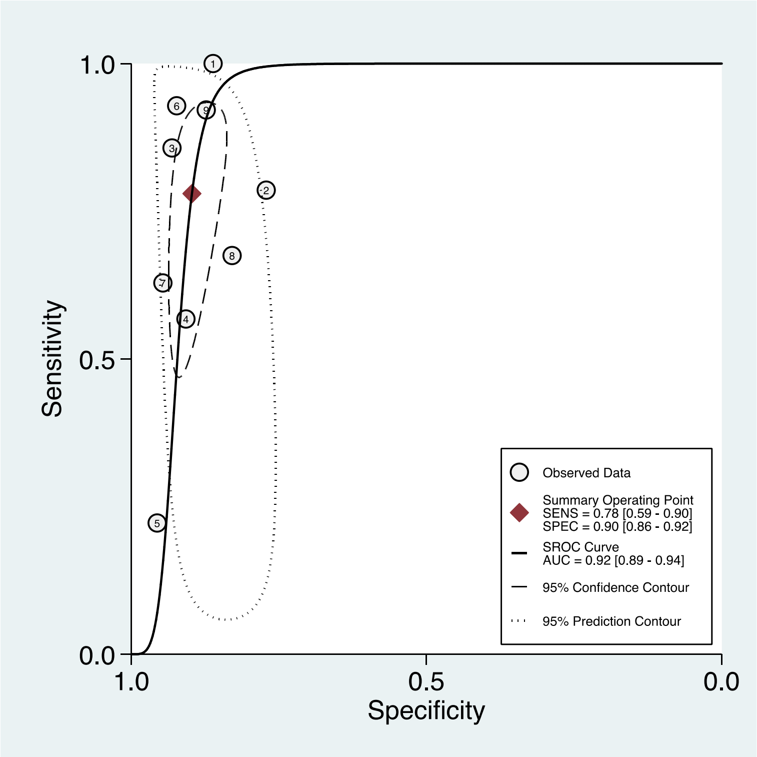

(F) Funnel plot

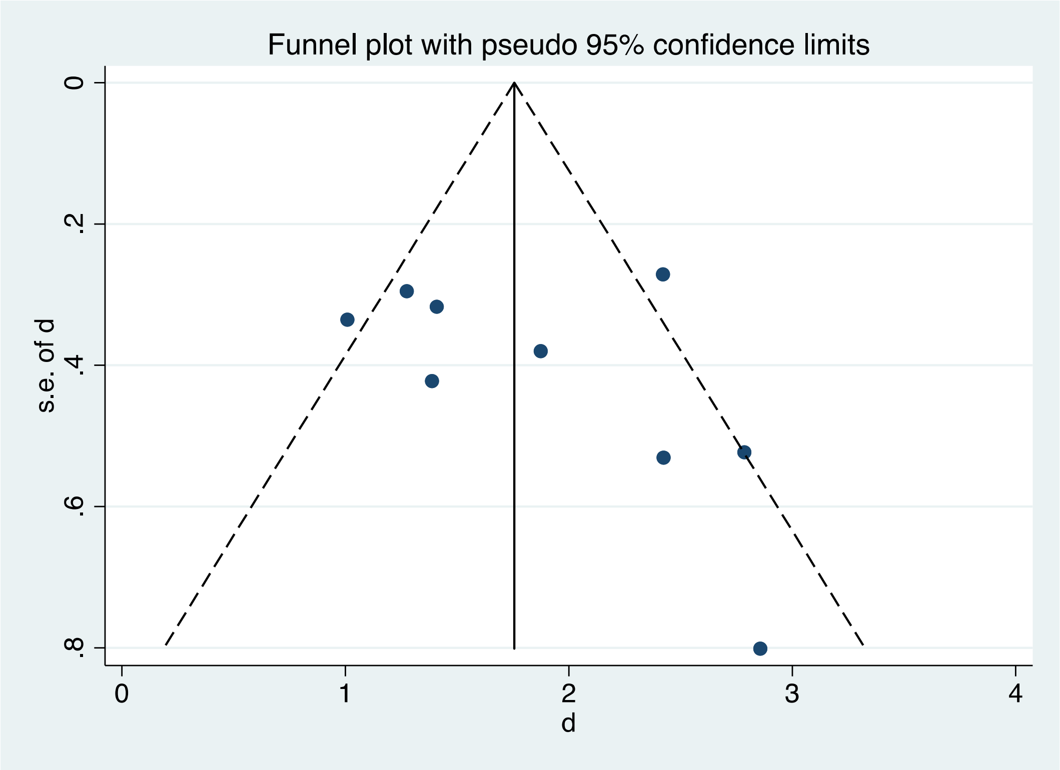

(G) Deeks funnel plot

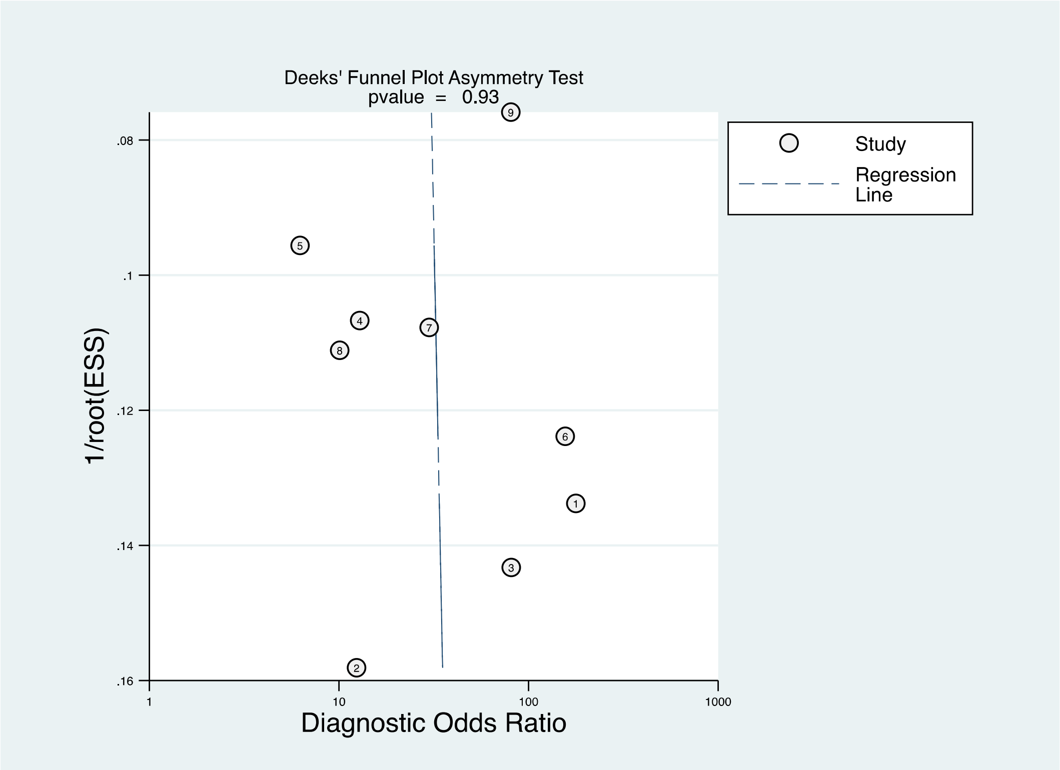

(H) Trim and fill method analysis

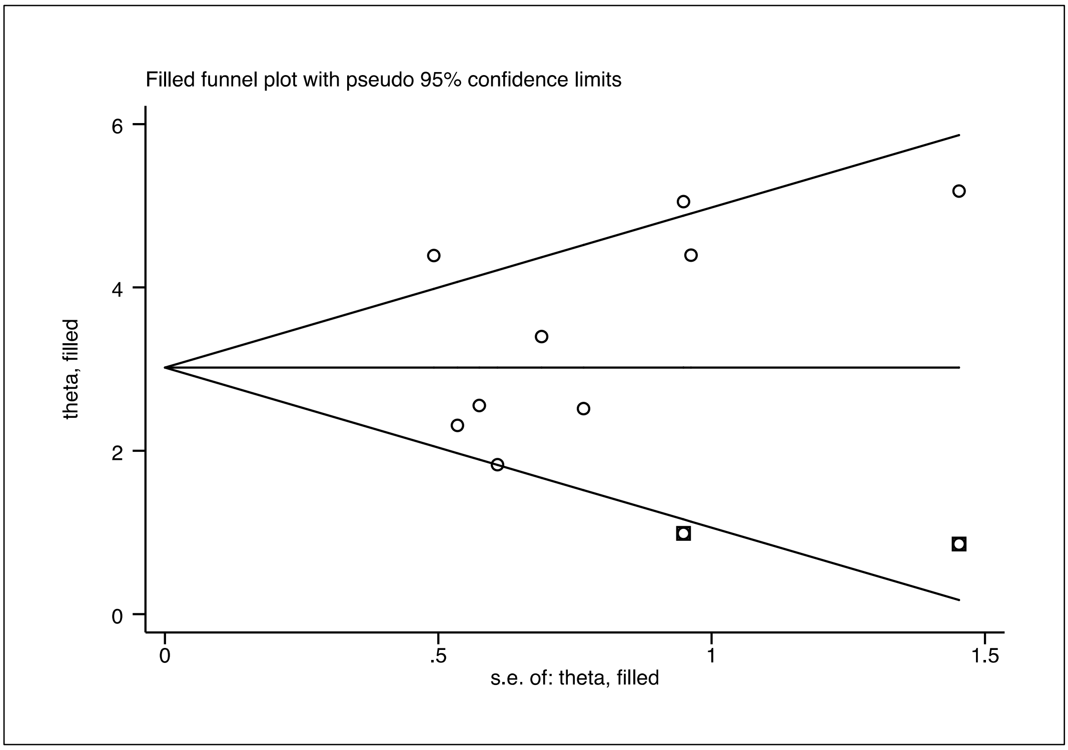

Filled funnel plot with pseudo 95% confidence limits
6
4
2
0
theta, filled
0
.5
1
1.5
s.e. of: theta, filled

Supplementary Figure S2 Meta-analysis of diagnostic performance for Node-RADS ≥ 4 as positive

(A) Forest plot of sensitivity and (B) Forest plot of specificity

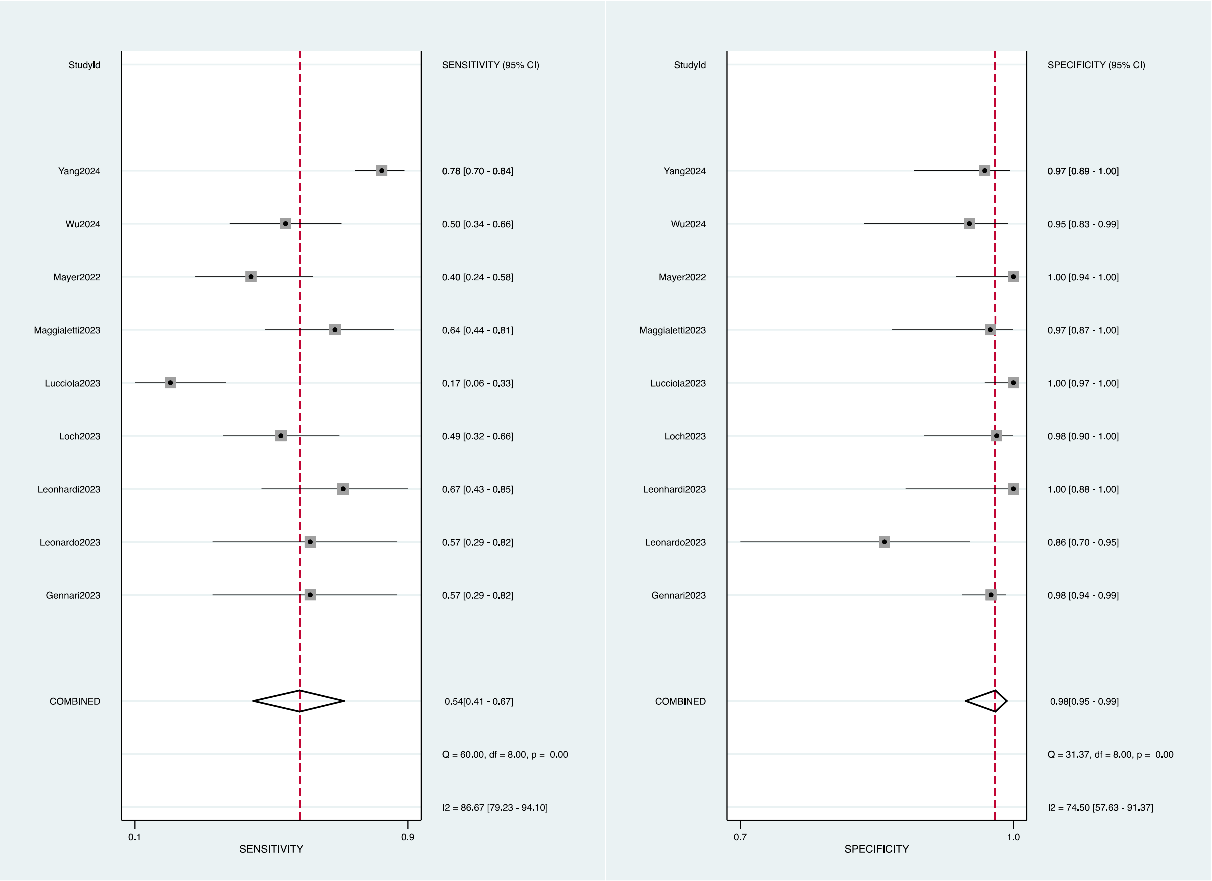

(C) Forest plot of positive likelihood ratio and (D) Forest plot of negative likelihood ratio

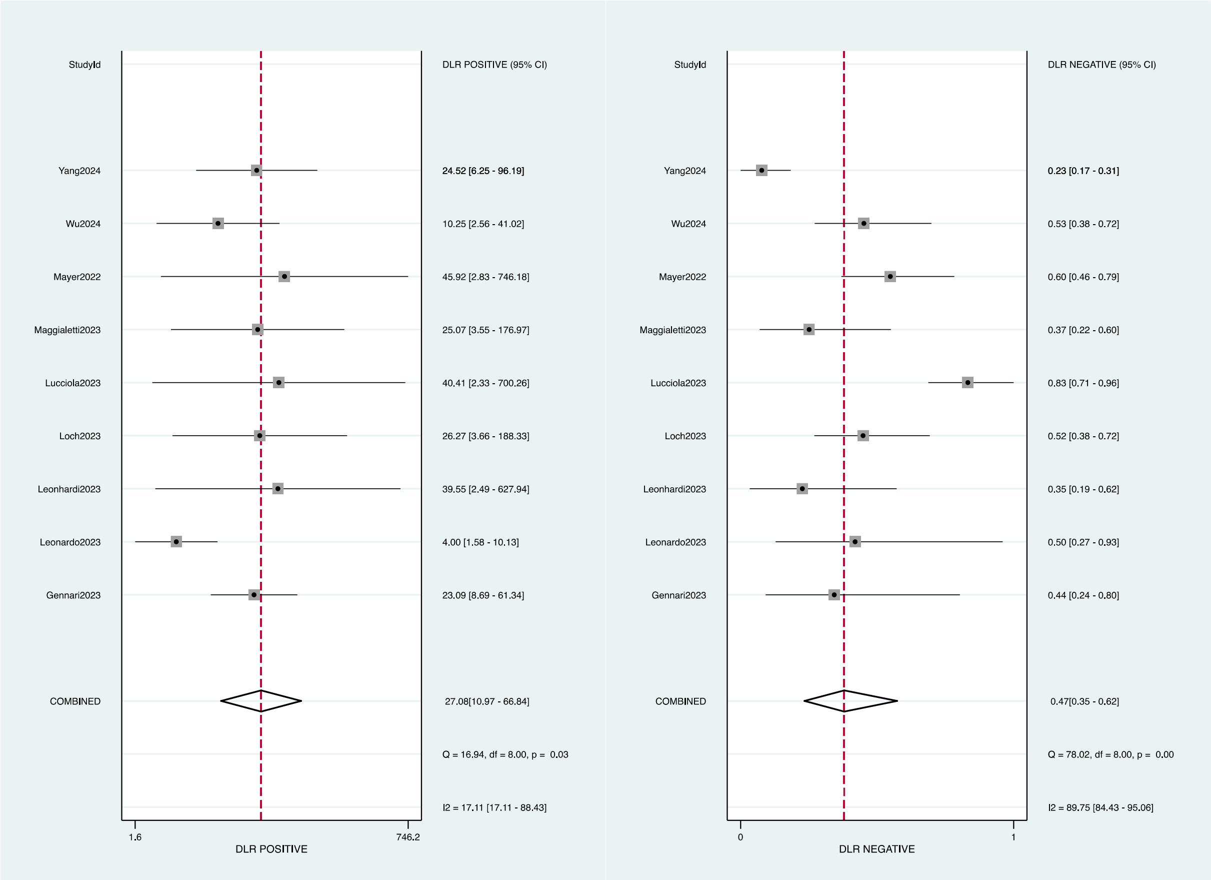

(E) Summary receiver operating characteristic curve

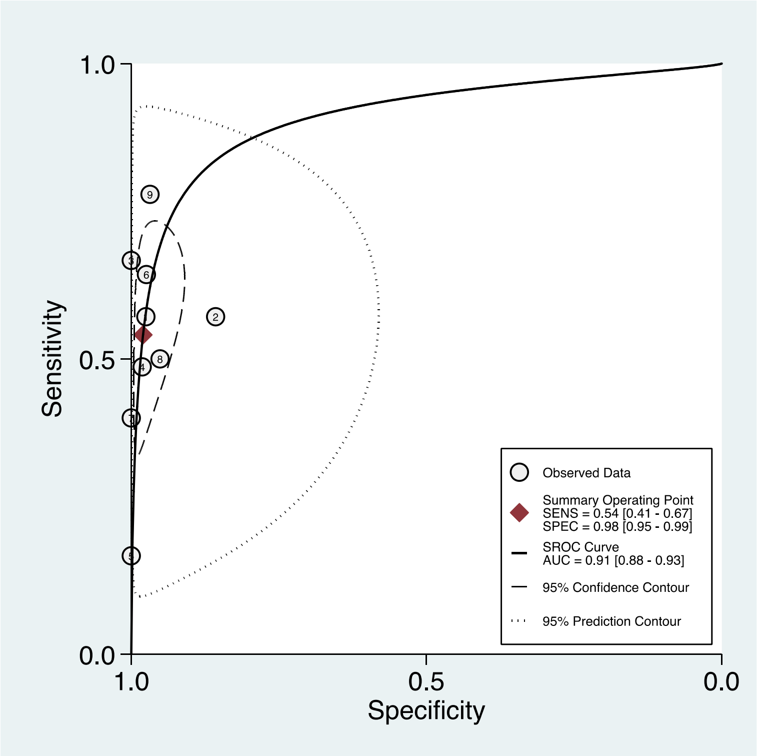

(F) Funnel plot

Eur Radiol (2024) Zhong JY, Mao SQ, Chen HD, et al.

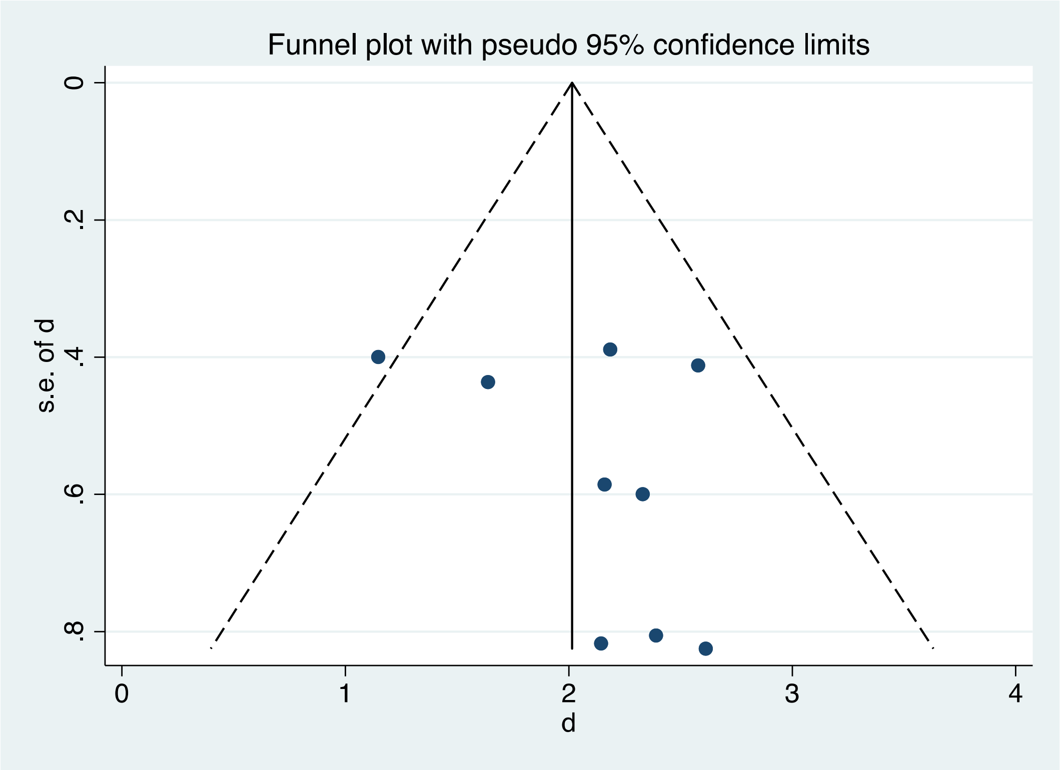

(G) Deeks funnel plot

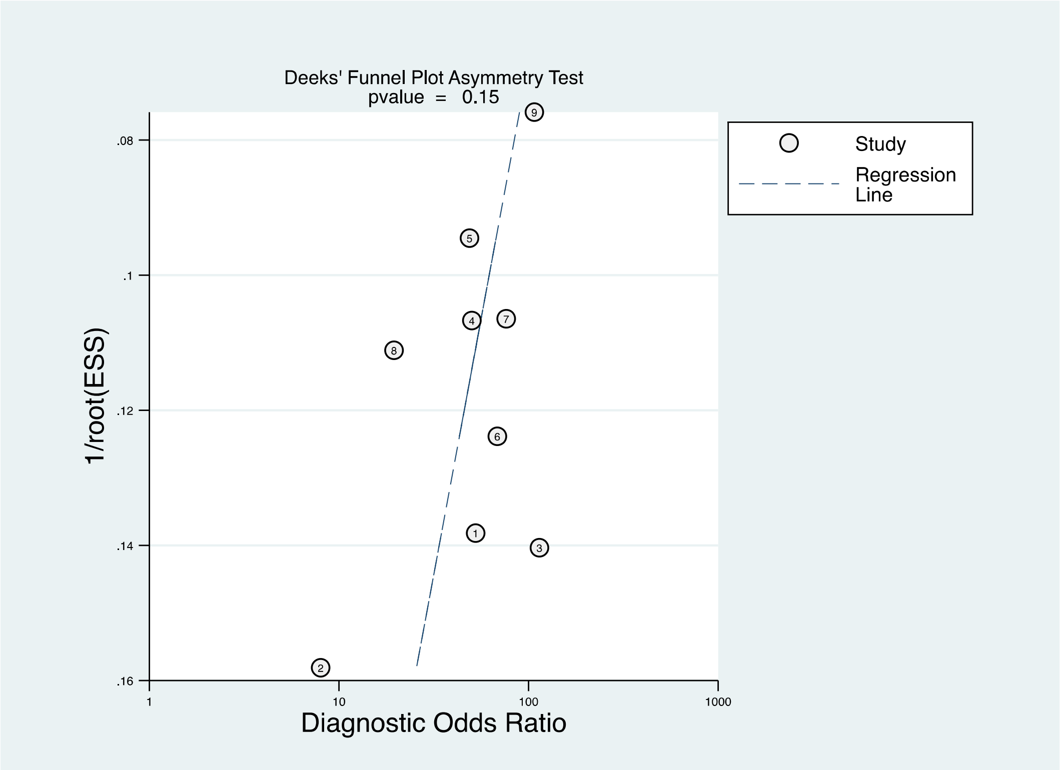

(H) Trim and fill method analysis

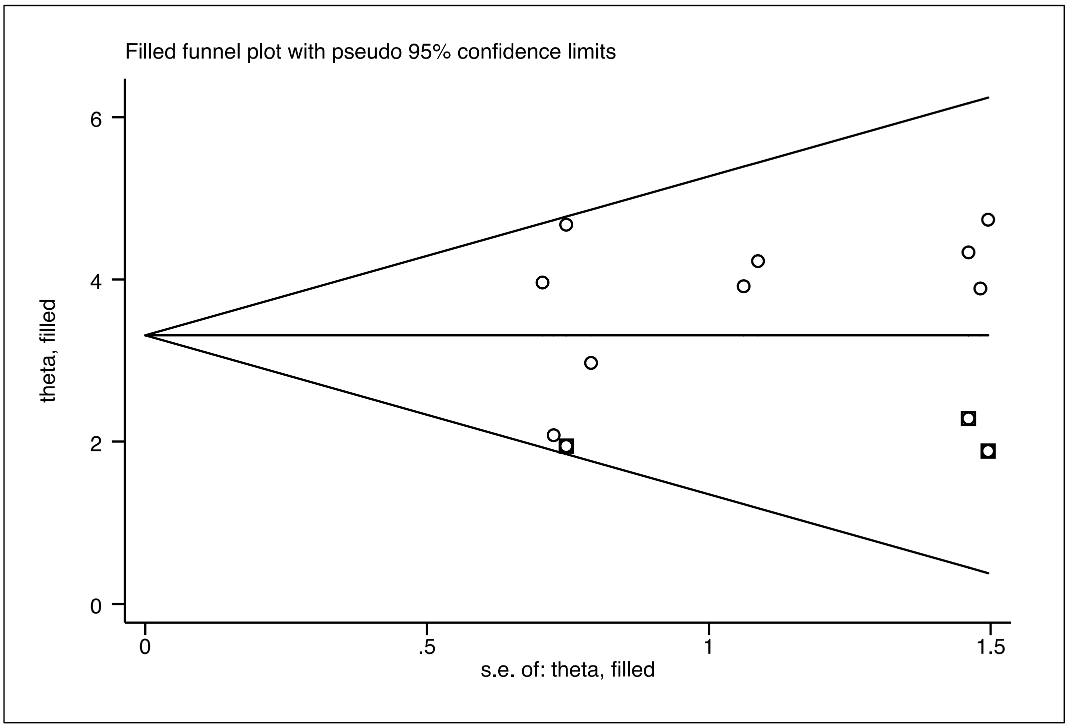

Eur Radiol (2024) Zhong JY, Mao SQ, Chen HD, et al.

Supplementary Figure S3 Forest plots of pooled category-wise malignancy rates

(A) Forest plot of malignancy rate of Node-RADS-1

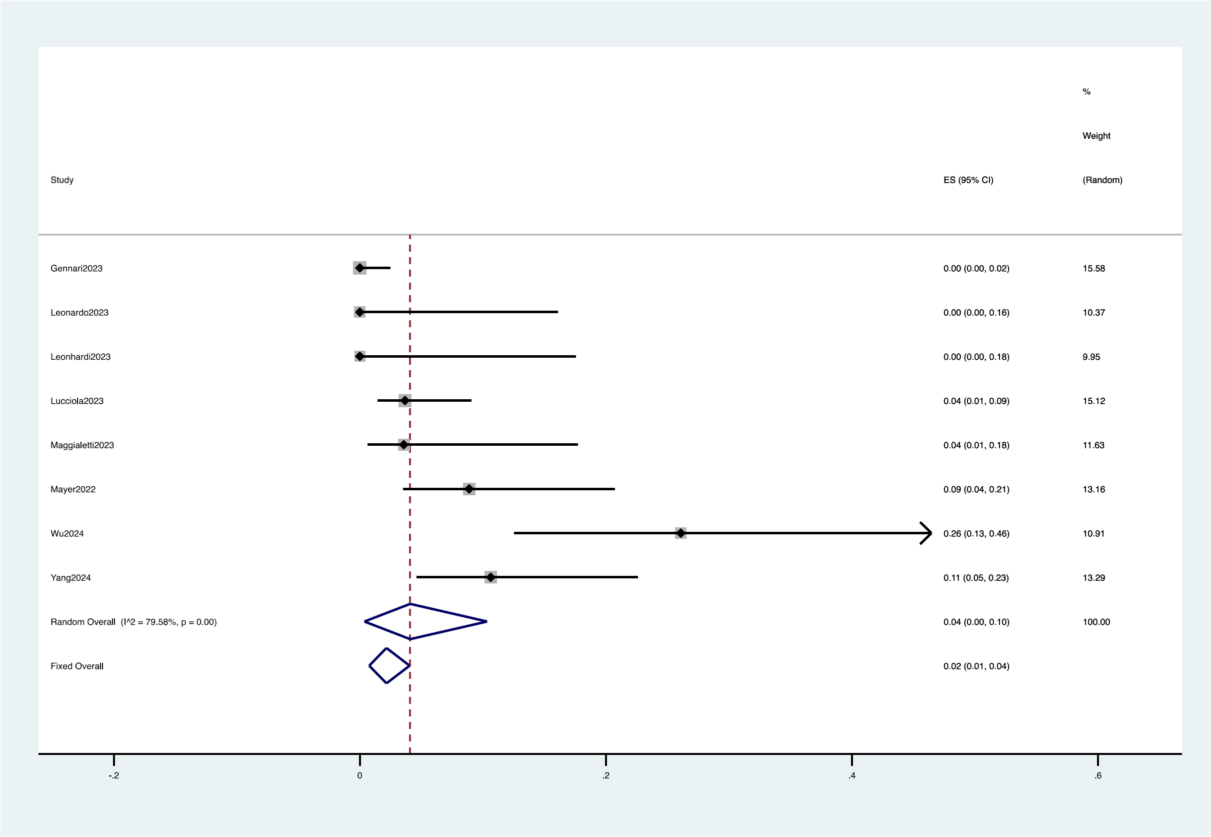

(B) Forest plot of malignancy rate of Node-RADS-2

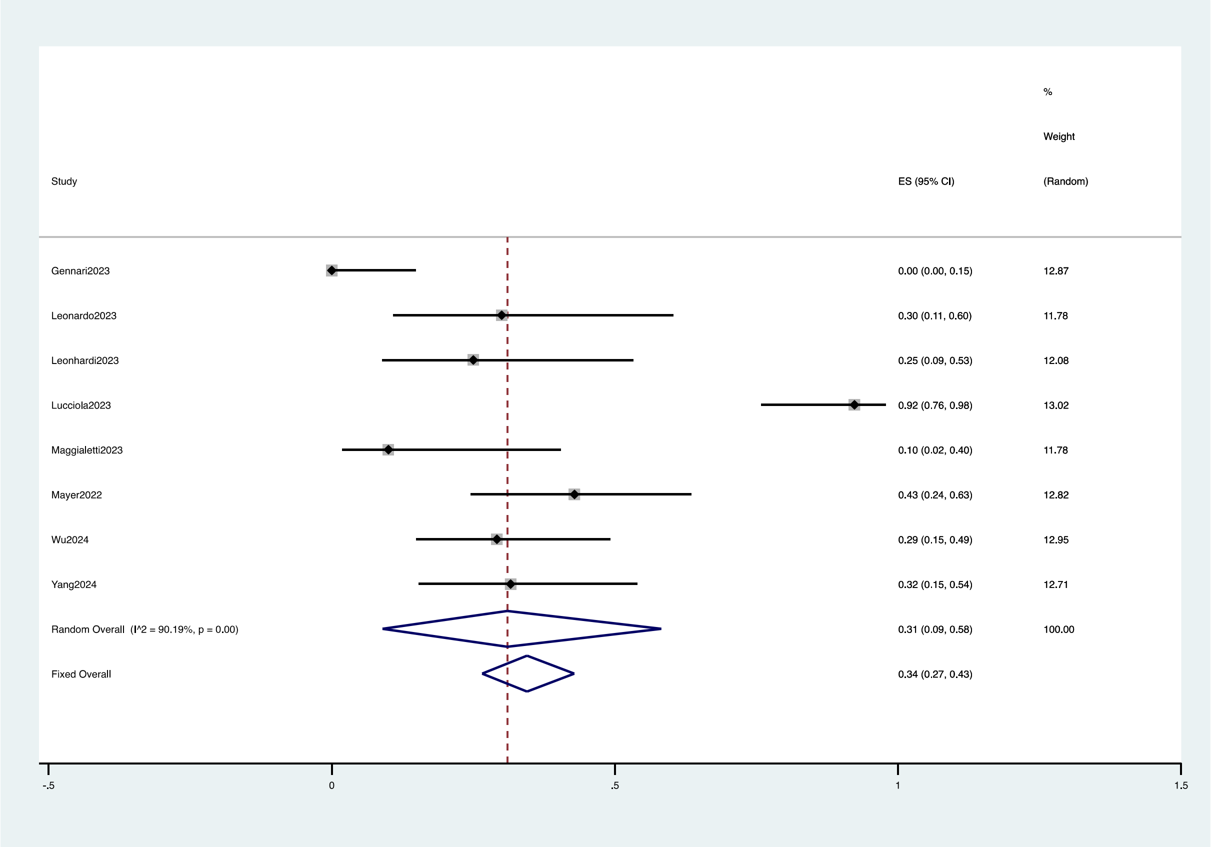

(C) Forest plot of malignancy rate of Node-RADS-3

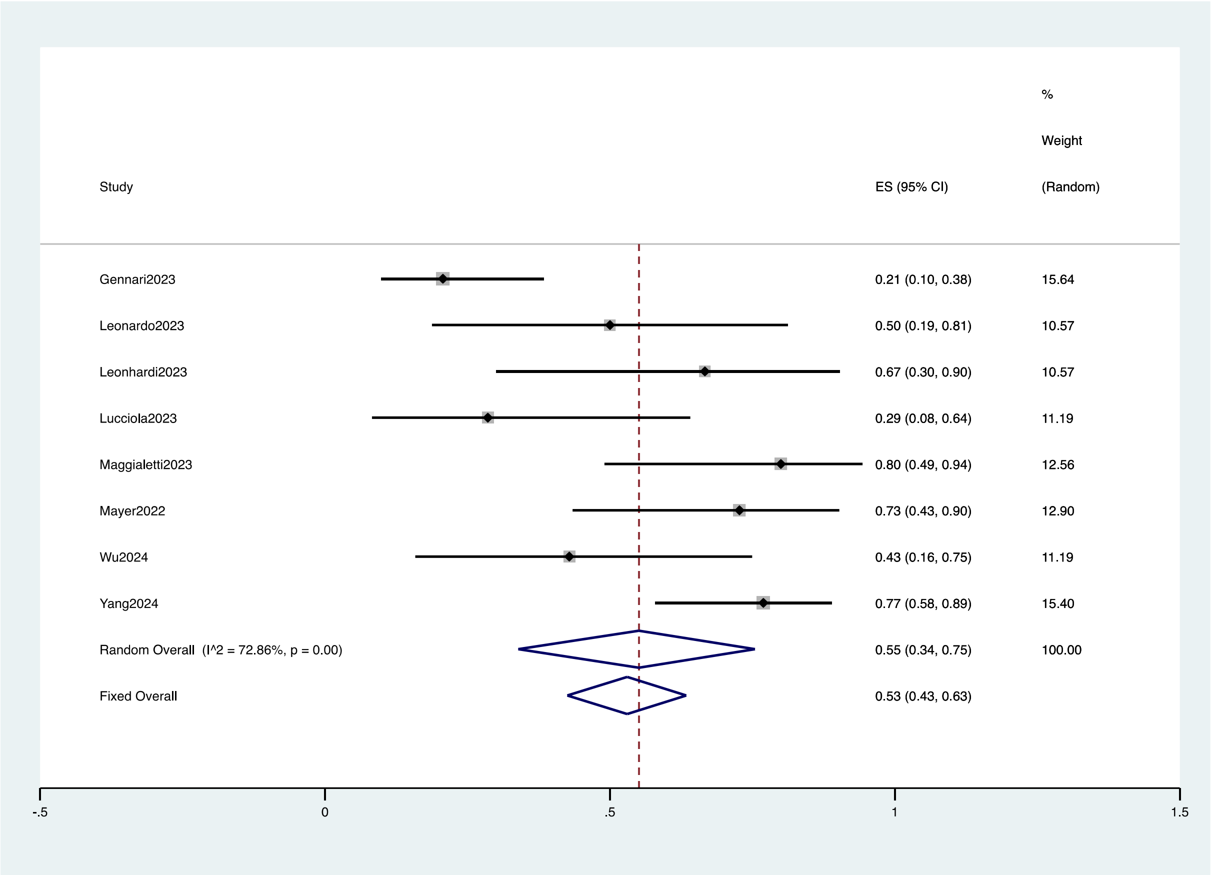

(D) Forest plot of malignancy rate of Node-RADS-4

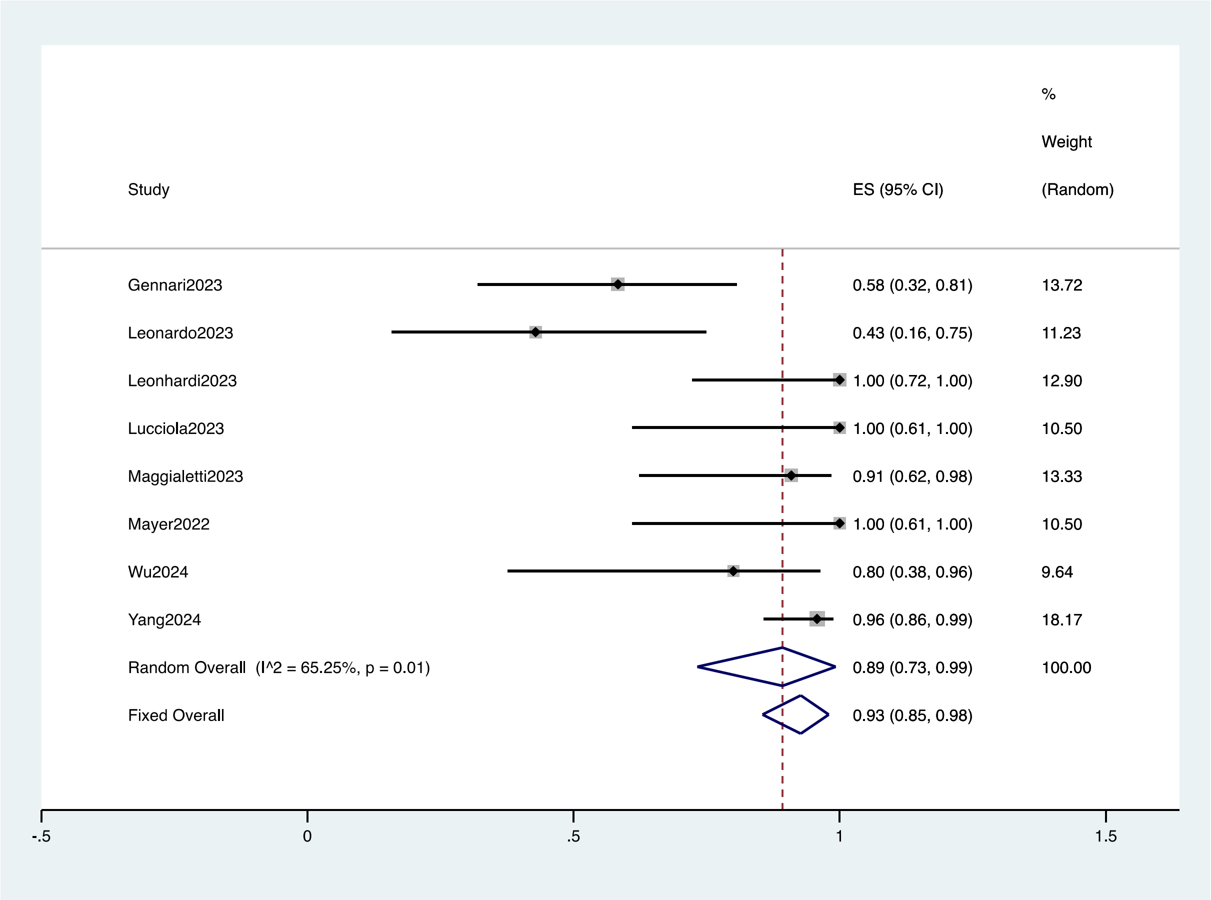

(E) Forest plot of malignancy rate of Node-RADS-5

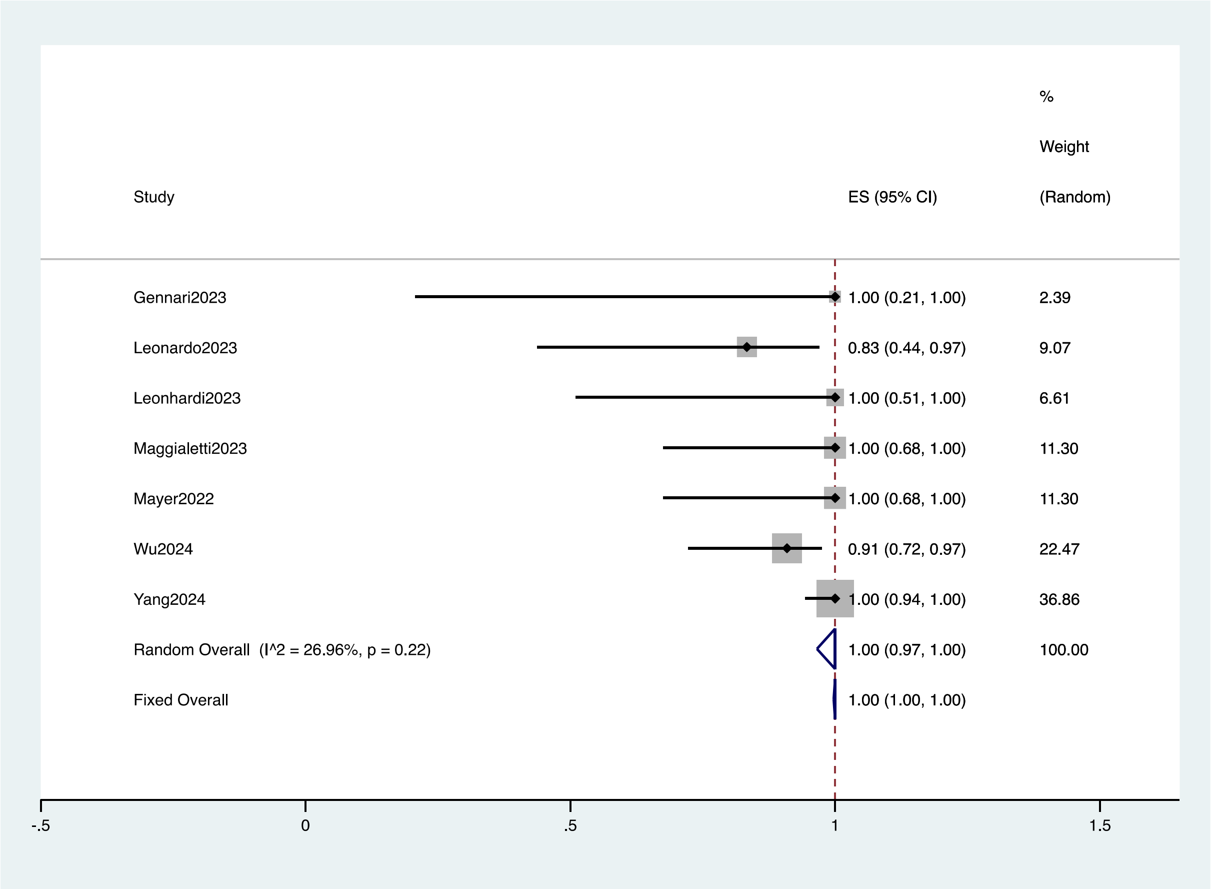

Supplement: Supplementary file 1 — ELECTRONIC SUPPLEMENTARY MATERIAL [file 330_2024_11160_MOESM1_ESM.pdf]
